# Factors Determining Epithelial-Mesenchymal Transition in Cancer Progression

**DOI:** 10.3390/ijms25168972

**Published:** 2024-08-17

**Authors:** Paulina Tomecka, Dominika Kunachowicz, Julia Górczyńska, Michał Gebuza, Jacek Kuźnicki, Katarzyna Skinderowicz, Anna Choromańska

**Affiliations:** 1 Faculty of Medicine, Wroclaw Medical University, 50-367 Wroclaw, Poland; tomeckapaulina@gmail.com (P.T.); juliagorczynska@tlen.pl (J.G.); m.gebuza@gmail.com (M.G.); jacek.kuznicki@student.umw.edu.pl (J.K.); katarzyna.skinderowicz@student.umw.edu.pl (K.S.); 2 Department of Pharmaceutical Biochemistry, Faculty of Pharmacy, Wroclaw Medical University, Borowska 211a, 50-556 Wroclaw, Poland; dominika.kunachowicz@student.umw.edu.pl; 3 Department of Molecular and Cellular Biology, Faculty of Pharmacy, Wroclaw Medical University, Borowska 211a, 50-556 Wroclaw, Poland

**Keywords:** epithelial-mesenchymal transition, cancer, tumor microenvironment, cellular plasticity, regulatory factors

## Abstract

Epithelial-mesenchymal transition (EMT) is a process in which an epithelial cell undergoes multiple modifications, acquiring both morphological and functional characteristics of a mesenchymal cell. This dynamic process is initiated by various inducing signals that activate numerous signaling pathways, leading to the stimulation of transcription factors. EMT plays a significant role in cancer progression, such as metastasis and tumor heterogeneity, as well as in drug resistance. In this article, we studied molecular mechanisms, epigenetic regulation, and cellular plasticity of EMT, as well as microenvironmental factors influencing this process. We included both in vivo and in vitro models in EMT investigation and clinical implications of EMT, such as the use of EMT in curing oncological patients and targeting its use in therapies. Additionally, this review concludes with future directions and challenges in the wide field of EMT.

## 1. Introduction

The epithelial-mesenchymal transition (EMT) is a crucial process in which epithelial cells acquire characteristics typical of mesenchymal cells [1,2]. In the initial epithelial phase, cells exhibit stable connections, and they maintained apical-basal polarity and active interactions with the basal membrane [3]. The conversion of epithelial cells to mesenchymal cells involves profound phenotypic changes [4]. During EMT, epithelial cells assume a loosely organized mesenchymal or fibroblast-like phenotype with features such as decreased intercellular adhesion, loss of apical-basal polarity, increased motility and invasive capacity, enhanced resistance to apoptosis, and increased extracellular matrix (ECM) production [5]. EMT involves the downregulation of epithelial cell markers and the concurrent upregulation of mesenchymal cell markers, culminating in the acquisition of mesenchymal properties.

The transition from epithelial to mesenchymal cells, known as EMT, is a key process in both embryonic development and certain pathological states in adults. This process has been divided into three types, each playing a specific role in different biological contexts. EMT Type 1 contributes to embryonic processes such as gastrulation, neural crest formation, or heart development [6]. EMT Type 2 is associated with wound healing, tissue regeneration, and organ fibrosis [7]. Finally, EMT Type 3 programming is closely linked to cancer progression, serving as a pivotal event in the invasion and metastasis of epithelial cancer cells [8]. A deeper grasp of these distinctions among EMT types can enhance our understanding of their functions and significance across diverse biological processes. This comprehension is pivotal in crafting effective therapeutic strategies for treating a range of conditions, including cancer.

**Figure 1:**
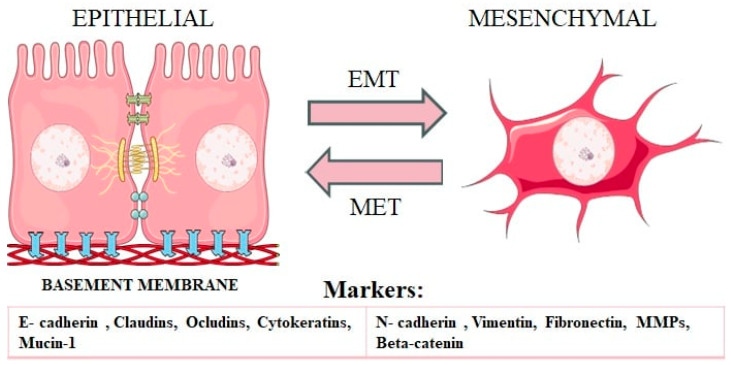
The image emphasizes the bidirectional transitions between epithelial and mesenchymal cell states, EMT and MET, highlighting distinct markers that characterize each state.

The process of EMT is reversible, with cells being able to revert to an epithelial state through mesenchymal-epithelial transition (MET). This transition from mesenchymal to epithelial states seems to play a crucial role in the metastatic spread of cancer cells [9,10] (Figure 1). EMT is, therefore, a dynamic process. It also leads to the creation of a series of intermediate cell states, in which cells exhibit a combination of epithelial and mesenchymal characteristics [11].

The tumor microenvironment significantly facilitates cancer metastasis and can trigger the occurrence of EMT [12]. The loss of E-cadherin expression, a tumor-suppressor protein, is frequently associated with EMT during cancer metastasis [13]. The expression of E-cadherin can be influenced by various experimental conditions and environmental cues. Therefore, the EMT paradigm, which relies on the loss of E-cadherin to determine the behavior and destiny of cancer cells, often remains a subject of ongoing inquiry [14]. Targeting factors related to EMT promise therapeutic interventions in various diseases, including cancer, fibrosis, and chronic inflammatory conditions [15,16]. Cancer cells undergoing EMT often acquire stem cell-like properties and exhibit resistance to apoptosis and cytostatics. Understanding the factors driving drug resistance associated with EMT can aid in developing strategies to overcome therapeutic resistance and improve treatment outcomes [17]. Additionally, factors associated with EMT can serve as diagnostic and prognostic biomarkers in cancer [18,19].

The EMT pathway is gaining increasing interest as a new therapeutic avenue in cancer treatment and can be targeted to prevent the spread of cancer cells in patients at early stages or to eliminate existing metastatic cells in advanced stages [20]. In regenerative medicine and tissue engineering, the knowledge of EMT regulators is essential for controlling cell behavior and directing tissue development for therapeutic purposes. The discussion on EMT concludes with an outlook on emerging bioengineering techniques that hold promise in revolutionizing our capacity to regulate EMT. These advancements could pave the way for innovative therapeutic interventions [21].

In this review, we have delved into the crucial role of EMT in cancer progression, highlighting the importance of understanding the factors that regulate this process. We have explored the molecular mechanisms of EMT, including microenvironmental influences and epigenetic regulations, shedding light on its significance in metastasis, drug resistance, and tumor heterogeneity. Furthermore, we have discussed the clinical implications of EMT, emphasizing its impact on disease outcomes and therapeutic strategies.

## 2. Molecular Mechanisms of EMT

### 2.1. Key Molecular Markers and Phenotypic Changes Associated with EMT

Commonly, regular epithelial cells form a tight multi- or single layer. The presence of numerous adherent proteins enables them to maintain this configuration and counteracts their mobility [22]. During EMT, static cuboidal cells change their morphology, as well as lose their organized positioning, acquiring a new fibroblast- or mesenchymal-like phenotype. They undergo numerous rearrangements, such as loss of apical-basal polarity, cytoskeleton modification, and a reduction of intercellular adhesion [23]. The expression of proteins, which are located on the surface of the cell, is changed, as well as the expression of cytoskeletal proteins. In the cytoskeleton, for instance, cytokeratin intermediate filaments are subjected to the substitution of vimentin, and the stress-fiber composition gains more actin. Modifications also affect the expression of microRNAs and ECM-degrading enzymes [24]. The loss of apical-basal polarity and basement membrane coherence occurs as a result of adherens junctions and desmosomes being disassembled [25]. These alterations cause the spindle shape of the cells and increase their mobility, which enables them to migrate and interfere with surrounding tissues. What is more, cells become more resistant to apoptosis [24].

EMT is stimulated by transcription factors like ZEB1/2, SNAIL1/2, and TWIST, among others. They can be used as markers to measure the progression of the EMT. The loss of epithelial marker expression with a parallel increase of mesenchymal factors is an EMT indicator [26]. The levels and activity of key epithelial molecular markers, such as E-cadherin, mucin-1, occludin, cytokeratins, desmoplakin, β-catenin, and α-catenin, are significantly lowered during EMT. On the contrary, mesenchymal markers being gained in the process of EMT are N-cadherin, vimentin, fibronectin, SNAIL (SNAI1), SLUG (SNAI2), and TWIST [24,26,27]. The stability of tight junctions is affected by the loss of claudins and occludins. They provide attachment to the other cells, like desmoplakin and E-cadherin, that are involved in building adherens junctions. The stability of tight junctions is affected by the loss of claudins and occludins. Normally, tight junctions provide attachment to the adherens junctions and actin cytoskeleton, as well as control the paracellular passage of ions and solutes in between cells and prevent the mixing of apical and basal protein membranes. The core of adherens junctions comprises cadherin and catenin protein family members. The adherens junctions play a role in initiating and stabilizing cell-cell adhesion, regulating actin cytoskeleton, intracellular signaling, and transcriptional regulation [28]. E-cadherin, an epithelial protein, functions as a tumor-suppressor gene and plays a crucial role in inhibiting cell invasiveness, regulating its polarity and differentiation, as well as limiting access to stem-like properties. It forms a part of the adherens junctions, where E-cadherin is linked to the actin cytoskeleton by α-, β-, γ-catenin, and protein p120. Through binding to adjacent cells, E-cadherin provides cell polarity [24]. It is responsible for regulating numerous signaling pathways sustaining epithelial phenotype and homeostasis of tissues. Lowering levels of E-cadherin may lead to the activation of EMT transcription factors and metastatic spread. During EMT, while E-cadherin is downregulated, N-cadherin is upregulated. N-cadherin is responsible for promoting angiogenesis and preserving the integrity of blood vessels [29].

Another mesenchymal marker is vimentin, an essential filamentous protein crucial in providing structural and functional support to the cell. As part of the cytoskeleton, vimentin is responsible for sustaining mechanical support, extracellular attachment, and central adhesion regulation. During EMT, this protein modulates genes for EMT inducers such as SNAIL, SLUG, TWIST, and ZEB1/2, as well as the key epigenetic factors. It also suppresses cellular differentiation and supports the cell’s self-renewability [30]. Also, fibronectin, an extracellular matrix glycoprotein, is regarded as an EMT marker. This molecule regulates cell attachment and migration and can be found in extracellular and plasma forms [31].

### 2.2. Role of Transcription Factors (e.g., SNAIL, TWIST, ZEB) in Regulating EMT

Factors stimulating EMT include the excessive expression of transcription factors, such as SNAIL1 and SNAIL2, TWIST, ZEB1 and ZEB2, FOX, and SOX. The SNAIL family plays a critical role as it influences the downregulation of E-cadherin, occludins, claudins, cytokeratin, and desmoplakin, as well as the upregulation of N-cadherin, fibronectin, collagens, and transcription factors such as TWIST and ZEB [27]. Each protein in the SNAIL family has a specific C-terminal domain, which consists of four-to-six C2H2-type zinc fingers. Through these structures, they link to the E-Box motif situated in target gene promoters. Of the three SNAIL proteins identified in the vertebrae, SNAIL1 and SNAIL2 have both been confirmed to play an integral role throughout EMT of all types. They recruit factors such as Polycomb Repressive Complex 2 (PRC2) to gain access to various transcriptional corepressor complexes and coordinate histone hypermethylation and deacetylation, which ultimately represses epithelial gene expression (e.g., their primary target E-cadherin, occludins, cadherin-16, and transcription factor 2 (TCF2)) to promote cellular detachment while increasing expression of mesenchymal genes (N-cadherin and ZEB1) and proteases to promote an invasive phenotype [5,20]. TWIST1, ZEB1, and ZEB2 also decrease the number of epithelial markers and increase the activity of mesenchymal ones. TWIST proteins regulate cadherin expression through recruiting SET8 histone methyltransferase, which induces histone 4 lysine 20 (H4K20) methylation. ZEB family decreases expression of the E-cadherin-encoding gene by influencing histone deacetylase. On the other hand, these transcription factors upregulate key mesenchymal marker- vimentin. Additionally, FOX proteins have an impact on EMT by regulating it oppositely. They increase the expression of genes encoding vimentin, fibronectin, and N-cadherin while downregulating the E-cadherin gene by overexpression of FOX proteins. Overexpressed FOXM1 also causes a rise of β-catenin amount. Other molecules undeniably vital for EMT are those comprising the SOX family, with SOX2 contributing to SNAIL expression upregulation [27].

### 2.3. Signaling Pathways Implicated in EMT Induction (e.g., TGF-β, Wnt/β-catenin, Notch)

EMT is stimulated by signaling pathways mediated by transforming growth factor- β (TGF-β)/bone morphogenic protein (BMP), Wnt–β-catenin, T-cell factor/lymphoid enhancer factor (TCF/LEF), Notch, Hedgehog, and receptor tyrosine kinases (RTKs). Plenty of stimuli from the local microenvironment, like growth factors and cytokines, hypoxia, and contact with the surrounding extracellular matrix, activate these pathways [32]. TGF-β is involved in angiogenesis, the stimulation of synthesis, and the degradation of ECM proteins, which function as a pivotal factor in cancer progression through ECM induction [27]. Proteins of the TGF-β family induce EMT through SMAD-mediated and non-SMAD signaling. Canonical TGF-β/BMP signaling is initiated by binding its ligands (TGF-β1, TGF-β2, BMPs) to the complex of Type I and II kinase receptors, activating SMAD2 and SMAD3. As they are phosphorylated, they bind to SMAD4. This induces translocation of the SMAD complex into the nucleus, where it represses or activates the target genes like SNAIL or SLUG [33]. TGF-β signaling is not only able to activate SMAD but inhibits it by activating SMAD6/7. Through binding to TGF-β receptor Type I, they directly prevent SMAD2/3 phosphorylation. Such inhibition can also be performed by SMURF, recruiting SMAD7 to the plasma membrane and linking to the receptor instead of R-SMADs (equivalent of SMAD6/7 in BMP) [32].

Besides SMAD-dependent signaling of TGF-β, there are various pathways that do not require SMAD. These are mostly seen in receptor tyrosine kinase signaling. For instance, oncogenic RTK activation of phosphoinositide 3-kinase/Akt kinase (PI3K/Akt) and Ras/Raf-signaling pathways are considered a hallmark of cancer EMT while combined with TGF-β signaling. The PI3K pathway hinders phosphorylation of β-catenin, which enables β-catenin to carry out the transcription of genes related to EMT. The Ras/Raf pathway encourages the activation of certain genes that support EMT. Additionally, TGF-β receptors (TGFRs) prompt the activation of p38 mitogen-activated protein kinase (MAPK), which subsequently triggers the activation of the EMT transcription factor FOXC2 [32].

In some cancers, abnormal Wnt signaling plays a significant role. While it is off, free β-catenin in the cytoplasm undergoes rapid phosphorylation and ubiquitination, leading to its degradation by the proteasome. However, upon Wnt binding to the frizzled receptor, the activity of the β-catenin destruction complex is halted by glycogen synthase kinase-3β (GSK3β), allowing β-catenin to move into the nucleus. Inside the nucleus, β-catenin activates the transcription of EMT effectors (like SNAIL and N-cadherin) through TCF/LEF. Moreover, LEF1 does not only trigger the expression of EMT effectors but also participates in the production of microRNAs associated with promoting EMT [32].

**Figure 2:**
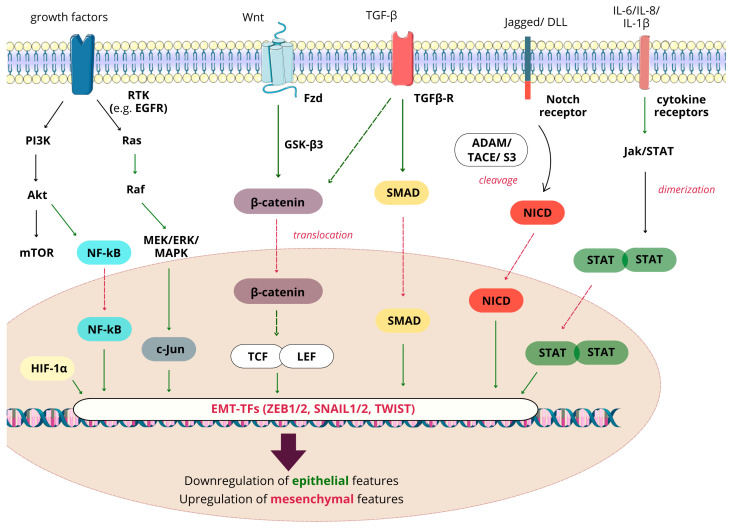
Representation of the key signaling pathways involved in EMT induction. See text for details.

The Notch-signaling pathway, participating in cell-to-cell communication and proliferation, can be considered as another key regulator in EMT induction. It can be activated by various stimuli, amongst which hypoxia, TGF-β, Jagged/Delta, fibroblast growth factor (FGF), or platelet-derived growth factor (PDGF) can be found. It is a juxtracine-signaling pathway where four transmembrane receptors require activation by ligands present on the surface of adjacent cells. Their interaction with the extracellular domain causes a split of Notch by tumor necrosis factor- α (TNF-α) converting enzyme (TACE)/ADAM protease. Subsequently, γ-secretase performs proteolytic cleavage resulting in the release of the Notch intracellular domain (NICD), which translocates to the nucleus. Afterwards, it links to the nuclear effector CSL, transforming its transcriptional repressor into a transcription activator complex. Thereby, NICD induces the expression of target genes, which promote EMT [34]. The schematic representation of pathways participating in EMT is presented in Figure 2.

## 3. Microenvironmental Factors Influencing EMT

### 3.1. Stromal Cells and Their Paracrine Signaling

Cancer cells forming solid tumors constantly communicate with surrounding cells and neighboring environments, ensuring their adaptation to unfavorable conditions. The tumor microenvironment (TME) comprises cells, both cancerous and non-malignant like immune or stromal cells, set in the complex ecosystem of ECM. The ECM serves as a non-cellular framework for cancer cells, accounting for the tissue shape and durability and providing regulators of cellular processes. Its macromolecular components are actively involved in adhesion, migration, proliferation, and differentiation. Therefore, it can promote tumor progression [35,36]. TME is rich in various types of cells with the prevailing number of fibroblasts, which have been termed cancer-associated fibroblasts (CAFs). CAFs can derive from various cell types, including mesenchymal fibroblasts, mesenchymal cells residing in bone marrow, transdifferentiated vascular endothelial cells, and adipocytes or stellate cells, as well as cells undergoing epithelial-mesenchymal transformation. As important cancer-progression mediators, CAFs are continuously generated from the above-listed cell types sent to the periphery of the tumor. Their role can be categorized in the release of growth factors enhancing cancer cell proliferation, promotion of neoangiogenesis, and participation in metastasis through EMT regulation, which had been recognized as their most critical contribution. It is based on the synergistic effect of the released growth factors and cytokines, affecting cancer cells in a paracrine manner [37].

CAF-derived factors include TGF-β, hepatocyte growth factor (HGF), FGF, stromal derived factor-1 (SDF-1), interleukins IL-6 and IL-32, and chemokine (C-C motif) ligand 5 (CCL5). In breast and bladder cancer, CAF-secreted TGF-β1 activates both SMAD-dependent and independent pathways, crucial for EMT. The SMAD pathway increases EMT- transcription factors (TFs) like SNAIL, SLUG, TWIST1/2, and ZEB1/2, while the non-canonical pathway activates MAPK/ERK, PI3K/Akt, and Rho GTPase. IL-6 from CAFs induces EMT through STAT3 in various cancers. Also, the binding of SDF-1 to the chemokine receptor CXCR4 promotes EMT and metastasis via the p38 kinase pathway, as shown in pancreatic and prostate cancer (PC) [38]. By secreting metalloproteinases MMP-2 and 9, CAFs also contribute to ECM remodeling, facilitating tumor cell dissemination. Moreover, MMPs themselves, along with increased collagen deposition in ECM, provide direct EMT-inducing signals [39]. Öhlund et al. differentiated CAFs into two types: myofibroblastic (myCAFs) and inflammatory (iCAFs). The former, residing close to the tumor, expressed a high amount of α-smooth muscle actin but a low number of cytokines. In contrast, iCAFs, located further, overexpressed IL-6 [40]. Co-culturing colon cancer organoids with an iCAF population led to ZEB1 upregulation and partial EMT, while cultures with myCAFs did not reproduce this effect [41].

Cancer-associated adipocytes (CAAs) are also a part of ECM. Their presence stems from the fact that cancer cells recruit adipocytes more effectively than normal tissues. Through adipocyte-cancer cell cross-talk, the phenotype and function of adipocytes change. For example, adipocytes/macrophage fatty acid-binding protein 2 (Ap2) and fatty acid-binding protein 4 (FABP 4) expression is lowered with an increased release of MMPs and inflammatory cytokines such as IL-6, IL-1β, and TNF-α. Therefore, CAAs contribute to tumor growth and progression, significantly promoting a proliferation of cancer cells and boosting their invasiveness. It has been shown that co-culturing mature adipocytes with breast cancer (BC) cells enhances cancer cell growth and EMT by increasing the expression of FOXC2, TWIST1, and N-cadherin [42].

Immune cells, mainly macrophages, neutrophils, dendritic cells, and bone marrow- and myeloid-derived suppressor cells, are another important component of TME [43]. Apart from establishing an immunosuppressive environment, immune cells via the secretion of cytokines and chemokines affect surrounding cancer cells in a paracrine manner. Meanwhile, cancer cells, in order to maintain immune suppression, secrete factors attracting more regulatory T lymphocytes, macrophages, or myeloid-derived suppressor cells (MDSCs) [38]. Among immune cells present in the TME, tumor-associated macrophages (TAMs) are largely involved in the process of EMT, promoting it on various levels. TAMs present a high level of similarity to M2-type macrophages, known for their role in cancer cell proliferation and invasion. In fact, TAMs comprise a collection of both M1 and M2 phenotypes, with the former prevailing at the onset of cancer and the latter dominating in its late stages [44]. Interactions between TAMs and cancer cells present in the tumor mass are mostly mediated by cytokines and growth factors secreted by the former, including potent EMT contributors or inducers such as TGF-β, TNF-α, IL-6, and IL-8, inducing multiple signaling pathways promoting mesenchymal markers expression: TGF-β/SMAD, Wnt/β-catenin, PI3K/Akt, and nuclear factor κ-B (NFκB) [43]. The synthesis and secretion of proteases by TAMs, including MMPs and cathepsins, significantly alter ECM, facilitating its remodeling and enhancing the disruption of the basement membrane. As a result of cathepsin activity, collagen fibers are degraded and their turnover occurs, while at the same time, TAM-derived cross-linking lysyl hydroxylase enzymes increase matrix stiffness [45]. What also closely links TAMs with metastasis, although not exactly EMT, is their participation in neoangiogenesis through vascular endothelial growth factor (VEGF) secretion and migration into vessels to assist in opening metastatic sites for cancer cells, known as the tumor microenvironment metastasis (TMEM) doorways [46].

Regarding neutrophils, high amounts of EMT-promoting TGF-β have been identified among the secreted factors in the example of lung cancer [47], along with MMPs, particularly MMP-9, and VEGF-upregulating cytokine oncostatin-M [48]. MMP-9 is also secreted by MDSCs, together with TGF-β and epidermal growth factor (EGF). Animal models suggest that EGF-activated EMT signaling can depend on CXCL5 chemokine induction [49]. It has been found that the stimulation of EMT by MDSCs additionally relies on the effect of IL-6 secretion, which activates the Janus kinase (Jak)/signal transducer and activator of transcription (STAT) pathway, as well as nitric oxide (NO), inducing Notch signaling in BC cells along with the maintenance of STAT activation [50]. Among other MDSC-derived factors, IL-28 as a target for interferon- λ (IFN-λ) and CCL11, a target for Akt/mTOR and Ras/MAPK/ERK, have been identified as EMT inducers [38]. The increased reactive oxygen species (ROS) production by MDSCs activate nuclear factor E2-related factor 2/antioxidant response element (Nrf2/ARE) signaling, while induced nitric oxide synthase (iNOS) triggers the cyclooxygenase-2 (COX-2)/β-catenin pathway; both processes can contribute to EMT [51].

As reported by Luo et al., BC cells attract MDSCs via CCL3, and in a positive feedback loop, these infiltrating MDSCs promote EMT via the PI3K/Akt/mTOR pathway [52]. More recently, secreted protein acidic rich in cysteine (SPARC) from MDSCs has been reported to have immunosuppressive and EMT-promoting properties [53]. Generally, MDSCs contribute to various steps of a metastatic cascade besides EMT induction and ECM reorganization, such as enhancing cancer cell intravasation, the survival of circulating tumor cells (CTCs), and their subsequent extravasation at the metastatic niche, the formation of which is also mediated by this type of immune cell [54]. Some evidence indicates that T lymphocytes, besides providing an immune response to present cancer cells, are able to induce EMT in cancer cell lines via produced cytokines, and it concerns both CD4+ and CD8+ T cells. For instance, CD4+ T-effector cells in pancreatic ductal adenocarcinoma initiated EMT through secreted IL-6 and TNF-α, presumably with the involvement of ZEB1 [55]. According to another report, CD4+ Th9 and CD4+ Th17, through secretion of IL-9 and IL-17, respectively, induced EMT in lung cancer cells. This finding was supported by studies on mice xenograft models [56].

### 3.2. Extracellular Matrix Components

An intrinsic network of ECM can contain over 300 different proteins, the exact composition and proportions of which vary in a cell- or tissue-dependent manner [57]. Since differences in structure and function, dictated by ECM composition, further determine its biomechanical and physical properties, ECM is the key player in modulating cells’ response to various stimuli and has been established as indispensable for all fundamental processes of cell biology. In cancers, ECM organization and regulation are often beyond spatiotemporal control, influencing the behavior of cells, signal transduction, and ligand-receptor interactions [58]. In the processes of tumor development, constant interactions between cancer cells and ECM occur. Alterations in ECM composition, organization, and interactions between its constituents and cancer cells are of major importance in facilitating cancer progression through formation of the metastatic niche [57].

The ECM is composed of a variety of macromolecules, capable of self-assembly and presenting different biochemical and physical properties: proteins, glycoproteins, proteoglycans, glycosaminoglycans, and polysaccharides, forming basement membrane and interstitial matrix present in the intercellular spaces [58]. Proteoglycans (PGs), present in the form of a hydrated gel, regulate the binding of cells to ECM, as well as endow tissue in mechanical resistance. Fibrous proteins comprise the second major group of ECM components, with an abundance of collagens, elastin, fibronectins, and laminins. Collagen, organized in triple helices, forms supramolecular structures of fibrils or networks, while elastin can assemble into cross-linked fibers and associate with collagen, allowing for an adaptation to mechanical stresses [57]. Fibronectin, through linkage to the integrins on the cell surface, is associated with the actin cytoskeleton, which is crucial for successful matrix formation; laminins are mainly responsible for cell adhesion. In addition, ECM provides a broad repertoire of other molecules, such as hyaluronic acid, integrins, growth factors, other cytokines, and MMPs [59].

There is a two-way relationship between ECM and EMT since it is both affected by this process as well as strongly involved in its occurrence. The metastasis-related ECM alterations can be manifested as cross-linking, rearrangements, and deposition or degradation of particular proteins present in the ECM [36]. Since EMT involves the disruption of the basement membrane, it is greatly dependent on its protein constituents and their interactions with polarized epithelial cells. Among these proteins, collagen directly interacts with cancer cell integrin and discoidin domain receptors (DDR). Such adhesions, mainly through Collagen-I (COLI) and Collagen-IV (COLIV), mediate cancer progression. Importantly, collagens have been observed upregulated in many cancers [60]. Colorectal cancer cells grown in vitro exhibit evident traits of EMT while cultured on collagen, along with increased clonogenicity and low differentiation, as shown by Kirkland et al. [61]. Similar patterns could be observed in BC cells of the MCF-7 line cultured on 3D collagen scaffolds, which exhibited upregulated mesenchymal markers and potentiated stemness [62].

Regarding the EMT-inducing role of COLI, activation of Rho signaling leads to cytoskeletal reorganization by favoring actin polymerization towards membrane protrusions and the disruption of adherens junctions [60]. Cancer cell adhesion to COLI induces the cadherin switch, requiring both integrin-β1 and DDR1 receptors. As demonstrated in pancreatic cancer cells, upon activation of integrins, the subsequent activation of downstream signaling is triggered, including focal adhesion kinase (FAK), FAK-related protein tyrosine kinase, Jun N-terminal kinase (JNK), and c-JUN. The outcome includes an enhancement of N-cadherin expression [63]. Moreover, in COLI-dependent N-cadherin induction, phosphorylation and the translocation of β-catenin are reported, leading to an induction of genes promoting proliferation and migration [64]. The effect of COLI-mediated E-cadherin repression is also indicated, and proposed mechanisms to explain this fact involve downregulation of the target gene expression engaging Src kinase and enhancing transcription of its repressors SNAIL and SLUG, which are regulated in a PI3K/Akt-dependent manner [65]. COLIV was also reported to interact with β1-integrin, with subsequent Src and extracellular signal-regulated kinase (ERK) phosphorylation, leading to an increase in cell motility. Furthermore, the migration of melanoma cells induced by the COLIV/β1-integrin pathway involves GTPases, PI3K, protein kinase C (PKC), and NF-κB [66].

Also, interactions with ECM components other than collagen can lead to EMT induction. Fibronectin-dependent integrin signaling contributes to EMT induction via activation of such pathways as FAK/Jak2/STAT3 and Src/ERK/PI3K, as well as upregulation of SNAIL, SMAD, N-cadherin, and vimentin. Upregulation of fibronectin expression, e.g., through TGF-β, was shown to be accompanied by the increase of EMT traits [36]. An interesting discovery concerning estrogen-related receptor α (ERRα) in BC cells was made. This molecule, due to the ability to bind to the fibronectin gene promoter, enhances cancer cell invasiveness and migratory capabilities [67]. Moreover, the binding of fibronectin to the cellular surface integrins facilitates growth factor receptor clustering, increasing the susceptibility of cells to their signaling [68].

Various isoforms of laminin, as another basement membrane protein, play their part in EMT as well. Laminin-mediated signaling can be transferred both through integrin and non-integrin receptors and affect multiple downstream signal mediators and pathway effectors, including MAPK, FAK, intracellular Ca^2+^, components of the cytoskeleton, and small Rho GTPases [69]. In the example of BC, it has been demonstrated that cancer cells express higher amounts of laminin than normal tissue, and such overexpression was reported to affect basement membrane integrity. Furthermore, an interaction with MMP-2 facilitates metastasis [70]. Stabilized laminin-5 and overexpressed laminin-332 inhibit the ubiquitination of Snail, maintaining EMT features and cancer cells’ metastatic ability [71].

**Figure 3:**
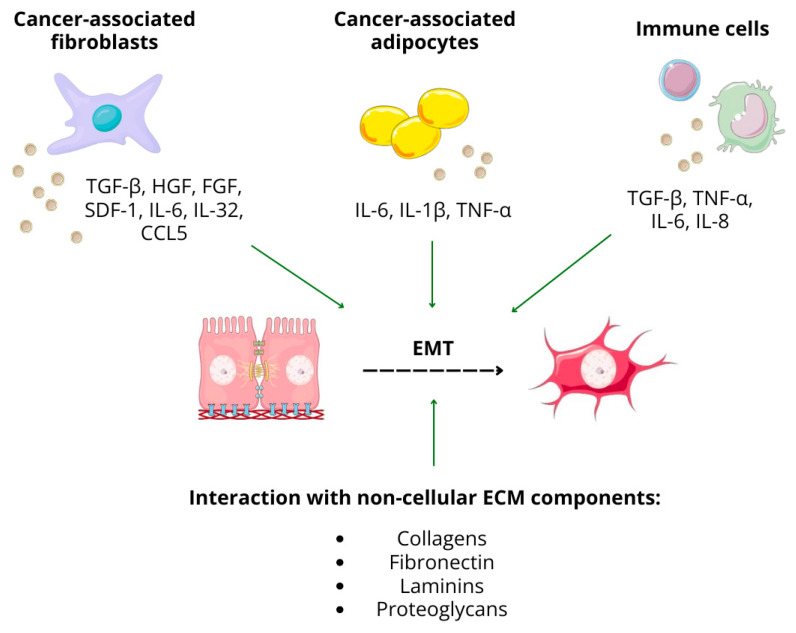
Soluble factors secreted by various cell populations, including CAFs, CAAs, and immune cells, can stimulate EMT. Also, the crosstalk between cancer cells and non-cellular components of ECM is an important contributing factor.

Members of proteoglycan superfamilies are involved in interactions between cells and cell-matrix ones. In general, PGs can confer oncogenic signaling and modulate cellular phenotype and functionality, but the expression of particular PGs differs among cancer types and varies depending on the clinical context. Some PGs are proposed to be associated with the epithelial, while others with the mesenchymal phenotype are listed among suggested EMT signatures [72]. In summary, ECM components influence central EMT transcriptional regulators, signaling pathways, and intracellular mediators, which are in turn highly responsive to ECM-derived stimuli in the dynamic cross-talk between ECM and cancer cells (Figure 3) [36].

### 3.3. Hypoxia and Metabolic Alterations in EMT

The spatial architecture of solid tumors, due to the accumulation of vast amounts of continuously proliferating cancer cells and irregular distribution of blood vessels formed as a result of neoangiogenesis, is often characterized by insufficient nutrient supply and poor oxygen diffusion. It leads to a significant decrease in oxygen levels in some parts of the tumor, known as hypoxia, which is common in cancers [73]. Depending on the oxygen partial pressure (pO_2_), two grades of hypoxia in cancer cells can be distinguished: intermediate hypoxia has pO_2_ values between 0.5 and 10 mmHg, severe hypoxia has a pO_2_ value lower than 0.5 mmHg, and healthy tissues normally contain a value of 10–80 mmHg [74]. Such a decrease of pO_2_ causes genomic instability and can lead to cell necrosis. Due to the indispensability of molecular oxygen in metabolic processes performed by mammalian cells, they have developed adaptive strategies to survive under differing oxygen concentrations, with hypoxia-inducible factors (HIFs) as key modulators of cellular response to its low levels. HIFs, which can be activated by a poor availability of oxygen, regulate the expression of multiple genes controlling viability, proliferation, energy generation, and other core activities of cells. Accordingly, an aberrant expression of these genes underlies multiple pathologies such as cancer [75,76].

Functionally, HIFs–HIF-1, 2, and 3–are heterodimers consisting of an oxygen-sensitive α subunit and constitutively expressed subunit β. HIF-1α is the factor that primarily accounts for cellular response to oxygen deprivation in the majority of tissue types [77]. In normoxia, HIF-1α is quickly degraded with a half-life in roughly 5 min due to proline residues hydroxylation by prolyl hydroxylases, von Hippel-Lindau protein (pVHL) factor binding, and following E3 ubiquitin ligase recruitment, which marks HIF-1α for proteasomal degradation. Additionally, the acetylation of lysine residue located in the oxygen-dependent degradation domain, performed by the arrest-defective-1 enzyme, is a modification recognized by the pVHL tumor suppressor, which is the second manner of tagging HIF-1α for ubiquitination [78]. Negative regulation of HIF-1α can occur also without the involvement of pVHL. Then, in the presence of a sufficient amount of oxygen, the asparagine-803 residue is hydroxylated by the enzyme known as factor-inhibiting HIF-1, preventing functional dimer formation [79].

Under hypoxic conditions, neither proline hydroxylation nor lysine acetylation is executed by the respective oxygen-dependent enzymes. Consequently, the HIF-1α structure is stabilized, and after translocation to the nucleus, it is free to dimerize with the β subunit, forming the active HIF-1 transcription factor. The following binding of HIF-1 to DNA sequences, known as hypoxia response elements (HREs) through basic helix-loop-helix domains, induces gene expression [80,81]. Moreover, HIF-1α can be activated also in an oxygen-independent manner as a result of the so-called oncogenic regulation. Growth factors, cytokines and numerous signal-transduction pathways activated during tumorigenesis, significantly contribute to the stimulation of HIF-1α and promote its accumulation in cancer cells in order to endow them with a potent pro-survival weapon to sustain proliferation in tumor microenvironment. Since the induction of hypoxia-related signaling is much more beneficial to cancer cells, mechanisms have been developed to enable the activation of HIFs even under sufficient tissue oxygenation [82]. Another major function of HIFs is to induce an expression of angiogenic factors as a response to low oxygen and nutrient supply to some tumor sites. Moreover, the subsequent expansion of intratumoral blood vessels provides a route for disseminated cancer cells to metastasize and invade other tissues, which is essential for cancer progression [83,84].

Supportively to these pro-survival functions, HIFs increase motility and, therefore, invasiveness of cancer cells, contributing to their metastatic spread and progression of the disease. HIFs modulate multiple signaling pathways driving the phenotypic switch in EMT and transcription factors regulating this process, as well as interfering with EMT-related non-coding RNA networks [82]. Stabilized HIF-1α activates TGF-β1, a major inducer of EMT, mediating the signal transmission via the TGF-β/SMAD pathway [85]. Upon binding to its receptors, which exhibit serine-threonine kinase activity, TGF-β1 forms a heterotetrameric complex able to phosphorylate SMAD2 and SMAD3. These core transcription factors after trimerization with SMAD4 are subjected to nuclear translocation, where the complex acts synergistically with other DNA-binding transcription factors, leading to the upregulation of TGF-β target genes [77,86]. As a result of the subsequent increase of EMT-related SNAIL, ZEB, and TWIST transcription factor expression, cellular epithelial markers are downregulated in favor of mesenchymal markers [87]. Moreover, TGF-β1 via SMAD reduces the activity of HIF-ubiquitinating proline hydroxylases, resulting in constitutive activation of HIF-1α through a vicious cycle of the regulatory loop [77]. TGF-β hypoxic signaling can be activated also in an SMAD-independent manner, including extracellular signal-regulated kinase1 and 2 (ERK1/2), PI3K/Akt, c-JNK, p38 MAPK, and Src tyrosine kinase pathways.

A considerable body of research associates the oncogenic Notch-signaling pathway with HIF-related EMT in a variety of mechanisms. Activated Notch1 facilitates the interaction between HIF-1α and the lysyl oxidase (LOX) gene promoter, with its product mediating the stabilization of the SNAIL1 protein and increasing its potency in EMT induction. Notch was also found necessary for EMT initiation following the hypoxia-mediated SNAIL and SLUG upregulation [88]. Integrative crosstalk between Jagged-1/Notch- and TGF-β/SMAD-signaling pathways has been established through HEY1. This specific downstream effector of the Notch pathway was found implicated in the TGF-β-mediated EMT [89,90,91]. Interestingly, the intracellular domain of the Notch1 receptor has a higher affinity to the HIF asparaginyl hydroxylase than HIF-1α itself. Therefore, Notch activation is translated into an increase of HIF activity. Such synergy contributes to the stimulation of EMT [92].

Stimulation of Wnt canonical cascade signaling is also established to accelerate EMT-mediated metastasis [93]. Hypoxia-mediated modulation of Wnt through the upregulated PI3K/Akt pathway occurs with post-translational β-catenin stabilization [94]. The interaction between HIF-1α and β-catenin has been found accountable for promoting EMT via HIF-1α activation and upregulation of canonical Wnt pathway-mediated signaling. On the hepatocellular carcinoma cell line, it has been shown that higher HIF-1α activity was correlated with an escalation of EMT features, while in the absence of this factor, EMT was reversed [95].

Another pathway activated in the hypoxic environment is nuclear factor κ-light-chain-enhancer of activated B cell (NF-κB) signaling, which also contributes to EMT induction and invasive cancer cell phenotype maintenance via upregulating TWIST1 expression [96]. Owing to the key importance of this transcription factor in triggering cellular stress response, the notion that it is activated by decreased oxygen availability is hardly surprising [97]. Hypoxic conditions promote degradation of the NF-κB inhibitor, resulting in its overactivation. HIF-1α-induced NF-κB has been shown to co-operate with ezrin, EGF, Ras proteins, and TGF-β in driving EMT. The PI3K/Akt cascade, together with TNF-α/NF-κB, was also found implicated in this process. However, the exact molecular grounds for these observations are not yet clear [98].

Several additional mechanisms are suggested to underlie hypoxic stimuli-mediated NF-κB induction. The evolutionarily conserved transforming growth factor-activating kinase 1 (TAK1)/IκB kinase (IKK) axis is considered a major activator of the NF-κB pathway and expression of its target genes [99]. There is also a connection between proline hydroxylases and NF-κB, which, along with other components of this pathway, has been identified as their substrate. Hence, the inhibition of these enzymes in hypoxia increases NF-κB signaling [97]. Moreover, hypoxia’induced NF-κB is able to regulate the expression of MMPs and SDF-1 [100]. All of the above may in consequence enhance EMT.

### 3.4. Metabolic Alterations

The ability of tumors to metastasize is, on several levels, associated with cancer cell metabolism. It is becoming clear that EMT induction is connected with changes in glucose, lipids, nucleotides, and amino acid metabolic pathways, which is a broadly investigated topic. Also, the fact that epithelial and mesenchymal cells are characterized by different energetic and metabolic requirements comes into play [101].

#### 3.4.1. Glucose Metabolism

Reprogramming glucose metabolism from mitochondrial oxidative phosphorylation towards enhanced aerobic glycolysis, even in the presence of oxygen, is a well-established cancer hallmark displayed by the majority of cancer cells. This glycolysis-dominant phenotype promotes the survival of cancer cells and tumor expansion due to increased glucose uptake and its conversion to lactate. Therefore, beyond satisfying the high energy demand of cancer cells in rapid ATP production, the generated intermediate compounds support their requirements for biomolecule synthesis [102,103]. Another effect, beneficial for cancer cell survival, is the minimized formation of ROS [104].

An increase in glucose uptake relies greatly on the increased expression of its transporters, mainly HIF-1α-activated GLUT1 and GLUT3. These two transporters are considered to have the worst prognostic value for cancer patients. An overexpression of GLUT1 correlated with an upregulation of vimentin and N-cadherin in laryngeal cancer, as reported by Zuo et al. [105]. Moreover, this transporter is suggested to be implicated in the maintenance of laryngeal cancer stem cells (CSCs) [106]. GLUT1 is also involved in upregulating MMP-2, as found in some cancer types, and correlates with their invasiveness [107]. In addition, high glucose levels were determined to mediate GLUT4-mediated VEGF secretion, along with EMT-related gene expression and increasing invasiveness of uterus endometrial cancer [108].

Another factor participating in glucose metabolism, upregulated in cancer cells, is hexokinase 2 (HK2), an enzyme catalyzing the first stage of glycolysis, i.e., the phosphorylation of glucose to glucose-6-phosphate. Like GLUT transporters, HK2 can be activated in hypoxic conditions. Its EMT-promoting activity has been established as kinase independent since the underlying mechanism includes binding and sequestering GSK3β to be phosphorylated and inhibited by protein kinase A (PKA). As a consequence, it translates to an increase in the amount and stability of GSK-3β targets, one of which is SNAIL. An additional role of HK2 is mediating SNAIL glycosylation, leading to its stabilization since, in this state, it is not susceptible to phosphorylation by GSK3 [109]. Apart from HK2, the induction of EMT entails a lot of other glycolytic enzymes, such as aldolases, enolases, phosphoglucose isomerase, phosphofructokinase-1, and pyruvate kinase [103]. A positive regulation of genes encoding these enzymes and thereby glycolytic events can be performed by TWIST-1, a known EMT transcription factor [110].

Another aspect of glucose metabolism associated with EMT is intensified lactate production and release in the TME as a consequence of aerobic glycolysis. Lactate is responsible for acidic conditions in the extracellular environment [111]. The implication of lactic acid to oncogenic progression is based on a number of effects concerning basal membrane remodeling and regulating of signaling pathways. Through promoting the expression of collagenases MMP-2 and MMP-9, it increases the degradation of Collagen Ⅳ, Ⅶ, and glycoproteins, thereby disrupting the ECM. Lactic acid can mediate both the direct activation of TGF-β and acts at the transcriptional level, enhancing its expression and also inducing a secretion of IL-6. Moreover, through activating SNAIL, it contributes to further ECM remodeling. Regarding cellular signaling, lactic acid mediates the activation of TGF-β/SMADs, IL-6/STAT3, and Wnt/β-catenin; all of these findings indicate its pleiotropic role in EMT [111,112].

Metabolic adaptation of malignant cancers includes also upregulation of the pentose phosphate pathway (PPP), which provides pentose phosphate, which is key for nucleic acids synthesis and NADPH. Parallel to glycolysis, PPP directs glucose to the oxidative branch of its catabolism. Therefore, both processes are strongly connected. The key enzyme catalyzing the first step of PPP, glucose-6-phosphate dehydrogenase (G6PD), has been reported to activate STAT3 and thereby promote EMT induction [113]. In turn, EMT inducers such as SNAIL and ZEB1 repress fructose-1,6-bisphosphatase 1 (FBP1) expression, directing glucose flux towards PPP. Another effect is the silencing of E-cadherin. Therefore, the invasiveness of cancer cells is increased and CSC features are acquired [114,115].

#### 3.4.2. Lipid Metabolism

Since lipids are indispensable components of all cellular membranes, involved in signaling, and serve as energy sources for the cells while deprived of nutrients, it is reasonable to assume that lipid metabolism alterations would be a factor affecting EMT. Indeed, its reprogramming has been reported to induce EMT and promote signal transduction in many EMT-related oncogenic pathways [116]. In the context of EMT, de novo lipogenesis, lipolysis, fatty acids oxidation, and lipid saturation are considered.

De novo synthesis of fatty acids is considered a characteristic feature of malignant cancers to satisfy the need for the production of membrane components [117]. Significant differences can be observed in lipdomic and proteomic profiles of epithelial versus mesenchymal cells. The former contain high amounts of monounsaturated fatty acids and are characterized by increased expression of the key enzymes coordinating de novo fatty acids synthesis, such as fatty acid synthase (FASN), acetyl-CoA carboxylase (ACC), and stearoyl-CoA desaturase (SCD) [118]. FASN has been found to activate VEGF and its receptor (VEGFR-2), mediated by EGFR/ERK axis; upregulation of this enzyme by TGF-β was demonstrated, leading to EMT [119]. Moreover, FASN increases the stability of lipid Rafts, affecting their composition and inducing the activity of the anchored proteins, phosphorylating E-cadherin, causing its degradation [117].

Regarding ACC1, its phosphorylation—which resulted in an increase of invading cells as a result of the TAK1 function—is connected with EMT induction through SMAD2 upregulation due to the increase in cellular acetyl-CoA levels [120]. In turn, SCD mediates the activation of Akt, which, through GSK-3β inactivation, further stabilizes β-catenin, thereby enhancing EMT promotion [121]. On the other hand, in mesenchymal-like cells, the lipogenesis rate is reduced in favor of triacylglycerol synthesis and the formation of lipid droplets. Studies also highlight the role of β-catenin in the regulation of lipid metabolism in cells of mesenchymal phenotype, which serves as a transcription modulator [118].

A low lipolysis rate, a process of degradation of triacylglycerol into glycerol and free fatty acids, is suspected to upregulate EMT-related genes, subsequently reducing differentiation of endothelial cells and suppressing E-cadherin expression [122]. Cancer cells, while deprived of key nutrients like glucose or amino acids, frequently utilize fatty acids as rich energy sources through β-oxidation [123]. In light of currently available evidence, fatty acid oxidation is a negative contributor to EMT since the accumulation of triglycerides favors EMT promotion [124]. Pro-oncogenic EMT signaling affects lipid metabolism as well through EGFR/MAPK, JNK, Jak/STAT, PI3K/Akt, Wnt, and TGF-β-associated pathways [117]. However, the underlying mechanisms and the exact contributions in lipid metabolism-EMT interplay are not yet comprehensively understood.

#### 3.4.3. Amino Acid Metabolism

Rapidly proliferating cancer cells exhibit an increased requirement for nitrogen necessary to perform biosynthetic processes. Since they are obtained from amino acids, they undergo quick degradation. Also, the demand for particular nonessential amino acids, exceeding intracellular supply, is a characteristic feature and induces the dependence of cancer cells on their exogenous sources. In order to provide sufficient amounts of amino acids, the upregulation of their transporters is induced, and an alteration in levels of enzymes catalyzing amino acid conversions occurs [125]. These enzymes are consequently involved in tumor progression and metastatic activity.

Glutamine (Gln) is regarded equally important as glucose in generating energy for cancer cells. A number of studies provided evidence that it is of key importance in the nucleotides and non-essential amino acid biosynthesis processes, as well as in the supply of substrates to the TCA cycle [126]. Glutaminolysis has been found to be intensified in cancers in general in order to increase the production of NADPH and maintain the efficacy of antioxidant systems [127]. The involvement of glutamine in EMT was indicated by studies evaluating the effects of glutaminase 1 (GLS1) inhibition, one of which turned out to be the repression of SNAIL. Conversely, the activation of this enzyme by TGF-β and/or Wnt has an EMT-promoting effect in a SNAIL-dependent pathway [128]. The deprivation of glutamine leads to a decrease in STAT3 phosphorylation, downregulating the expression of target genes, including those which are EMT-related [129]. STAT3-induced EMT has been shown to depend also on glutamine dehydrogenase, while downregulation is connected with a decrease in vimentin and ZEB1 expression in favor of E-cadherin [130].

The oncogenic metabolism of Gln is strictly connected with asparagine (Asn), and these two amino acids closely cooperate in tumor progression and metastasis. When the availability of Gln in the extracellular environment is restricted, tumor cells switch to Asn utilization with the purpose of protein synthesis and, consequently, maintenance of a high cellular proliferation rate [126]. Additionally, Asn promotes Gln biosynthesis via the upregulating expression of the key enzymes, suppresses apoptosis, and regulates the oncogenic mTOR pathway [131]. The link between Asn and EMT is supported by considerable evidence, such as high content of this amino acid in EMT-driving proteins, compromised EMT upon limited Asn bioavailability, and promotion of EMT-like disseminated cells survival in circulation [126]. It also underlines the role of asparagine synthetase (ASNS) in driving metastasis. Furthermore, Asn was demonstrated to mediate transcriptional regulation of TWIST and N-cadherin, but the underlying mechanism remains unclear [132].

In cancer cells, de novo serine synthesis from glucose is increased, even if the extracellular serine amounts are sufficient. Interestingly, serine is classified as the third-most-consumed nutrient by cancer cells and is easily converted into glycine used for nucleotide biosynthesis in the serine/glycine one-carbon (SGOC) pathway [133]. Apart from its role in glycine synthesis, serine is a key precursor of cysteine, glutathione (GSH), and ceramides; it also modulates NADPH/NADP+ redox balance and epigenetic regulation, strongly supporting proliferative activity of cancer cells. An increasing body of evidence highlights the active contribution of the serine synthesis pathway (SSP) to the phenotypic transition of cancer cells [134]. As shown in a number of studies, SSP is often upregulated in cancer cells. In addition, enzymes involved in SSP influence EMT and metastasis in mechanisms other than serine biosynthesis, such as ECM remodeling through the activation of MMPs and the downregulation of laminin-reducing cellular adhesion. The generation of formate and fatty acids in the course of SSP also complies with EMT induction, as well as upregulation of EMT marker genes [135].

#### 3.4.4. Nucleic Acids Metabolism

Contrary to healthy ones, cancer cells largely rely on the so-called nucleoside salvage, i.e., de novo nucleotide synthesis to provide constant delivery of deoxyribonucleoside triphosphates (dNTPs) to satisfy the needs of uncontrollably proliferating cells [136]. Along with pyrimidine degradation, it is controlled by a complicated enzymatic network referred to as pyrimidine metabolism (PyM), whose components can be activated by oncogenes [137]. It is now accepted that the catabolic activity of PyM enzymes is utilized by cells that undergo switches into a mesenchymal phenotype to maintain it [137]. The relationship between PyM and EMT is in fact two-way–overexpression of EMT-TFs, such as ZEB1 or TWIST, which results in the accumulation of the pyrimidine degradation products. These are necessary in EMT, as shown in the example of dihydrothymine and dihydrouracil on BC cells with the prominent role of dihydropyrimidine dehydrogenase [138].

Purines present in the extracellular space can generate signals, received by receptors in or near the cells, which is known as purinergic signaling and regarded as an underappreciated pro-metastatic factor [139]. It is associated with EMT induction through dephosphorylated ATP metabolites and upregulation of P2Y and P2X purinergic receptors, which, upon activation by UTP, promote SNAIL and TWIST expression and increase vimentin levels. ERK1/2 and PI3K/Akt pathways are also implicated in this process [140]. It has also been proposed that nucleosides may contribute to EMT induction by TGF-β [141]. Other reported effects of purinoceptor activation include MMP-9 expression and the induction of MAPK and NF-κB signaling [142]. In light of these findings, pyrimidine nucleotides have been proposed by Siddiqui et al. to be considered oncometabolites [137].

### 3.5. Inflammatory Cytokines and Growth Factors Promoting EMT Induction

#### 3.5.1. Inflammatory Cytokines

Inflammation is an important cancer hallmark, parallel to tumor development from the initial stages, through its progression, and towards the formation of metastases. Cancer-related inflammatory responses contribute to the maintenance of unlimited replicative potential of cells, enhance neoangiogenesis, induce immunosuppression, and augment tumor extravasation, supporting its spread [143,144]. Upon EMT induction, the ability of cancer cells to remain in a mesenchymal state depends on the presence and function of cytokines and growth factors, which have earlier participated in EMT activation [145].

#### 3.5.2. TNF-α

TNF-α is a cytokine of pleiotropic biological activities and of major importance in cancer progression through the modulation of processes such as inflammation, immune cell functionality, angiogenesis, cell proliferation without inducing their differentiation, and apoptosis [146]. Studies suggest its significance even in the initial stages of cancer development and promotion. The relevance of TNF-α is reflected in calling it a master switch, orchestrating a complex two-way relationship between inflammation and cancer. Constitutive secretion of TNF-α is observed in many cancer types. Since its receptors are expressed both on the surface of epithelial and stromal cells, TNF-α can modify TME to enhance cancer development [147]. Also, the innate immune cells present in the TME secrete cytokines, including IL-6, TNF-α, and IL-1, which, through recruiting more of the inflammatory cells, further potentiate this effect, contribute to cancer cell proliferation and survival [148].

Intense research established the role of TNF-α in cancer inflammation-mediated metastasis via increased expression of numerous factors, such as VEGF, FGF, EGFR, TGF-β, IL-8, or adhesion molecules, including the activation of JNK and AP-1 pathways [148]. A promotion of metastasis with TNFα involvement depends on NF-κB activation, which is a crucial step towards EMT induction and enables the stabilization of SNAIL by inhibiting its proteasomal degradation [149]. SNAIL, which remains in its non-phosphorylated, functional state, directs a cell into EMT.

However, NF-κB activation is not the sole contribution of TNF-α to EMT induction. Through inhibiting GSK-3β, it activates Wnt/β-catenin signaling, which further leads to the maintenance of the SNAIL functional state and its nuclear localization, as demonstrated in gastric cancer cells. Since SNAIL can induce the Wnt pathway, a positive feedback loop is generated [150]. The increased expression of TNF-α seems to be correlated with high levels of β-catenin and SNAIL at the tumor-stromal boundary of dissociating cancer cells [151]. Additionally, TNFα has also been shown to mediate the overexpression of MMPs, enhancing the migration of cancer cells and tumor expansion [152].

#### 3.5.3. IL-1β

Not only immune cells and fibroblasts, but also cancer cells are able to secrete interleukin-1β (IL-1β), which has been shown to be overexpressed in various solid tumors and linked to worse prognoses, promoting tumor growth and invasiveness. The contribution of IL-1β to disease progression is well established in several cancers, such as skin, breast, and ovarian cancer, while its implication in other types is less understood, with some studies producing contradictory results [153,154]. Besides mediating immune response, the roles played by IL-1β in cells include regulating gene expression, cytokine release, and modulation of cell adhesive and migratory properties, placing it in close association with EMT.

As discussed before, the current knowledge leaves no doubt that EMT and inflammation are highly interconnected. A role in these processes is also played by IL-1β, considered one of most potent cytokines [153]. IL-1β is an important mediator in TME shaping. Through activating FAK kinase and Src, it increases the expression of MMPs to enable cancer cell migration [155]. It was shown that tumor-derived IL-1β influences β1-integrin expression, enabling the adhesion of disseminated ovarian cancer cells to mesothelium and their subsequent implantation [156].

The interaction of IL-1β with its IL-1 Type 1 receptor (IL-1R1) activates β-catenin-dependent signaling, upregulating TWIST1 and SNAIL1 and triggering the EMT program in cancer cells. Transformation into the mesenchymal phenotype occurs also through NF-κb-induced STAT3 activation [157]. Moreover, IL-1R signaling through the expression of inflammatory mediators promotes cancer cell survival and stemness development (both IL-1β and IL-1α reprogram mesenchymal stem cells secrete β-catenin-inducing factors), creating a cancer stem cell niche [158]. Importantly, IL-1β is suspected to increase IL-6 secretion via a transglutaminase 2/NF-κB pathway, enhancing growth and aggressiveness, as presented in BC cells [159].

Studies on genome-wide mRNA expression patterns revealed an interesting correlation between IL-1β, chemokine CCL2 (also known as monocyte chemoattractant protein-1, MCP-1), and osteoprotegerin (OPG) in BC. The latter is a regulatory factor of bone resorption and remodeling processes from the TNF receptor superfamily, while CCL2 is a well-known cell migration and EMT promoter. IL-1β was shown to upregulate OPG expression in MAPK-dependent pathways and enhance its secretion by tumor cells, associated with macrophage infiltration [160]. It stands in line with the previous findings that IL-1β expression correlates with BC relapse and the occurrence of metastases to bones, and that IL-1β is abundantly secreted by specific bone-infiltrating mouse BC cell lines. Taken together, these results indicate that IL-1β/IL-1R1 interaction via OPG and CCL2 largely influences BC cell metastatic activity to bone, but the cellular context is crucial. The inhibition of IL-1β interaction with its receptor was observed to maintain the disseminated cancer cells in the bone microenvironment in a state of dormancy [161,162,163].

#### 3.5.4. IL-6

Interleukin-6, a glycosylated polypeptide of 25 kDa, is a major inflammatory cytokine. Besides supporting numerous physiological functions, it has been linked to tumorigenesis and cancer progression. Abundant in the tumor microenvironment, IL-6 is involved in DNA damage repair and cancer-related inflammation. Also, metabolic remodeling and resistance to apoptosis are cancer hallmarks to which IL-6 activity is attributed [164]. The pro-survival and proliferation-inducing functions are governed by the capability of this cytokine to activate PI3K/Akt, NF-κB, and MAPK/ERK pathways. Moreover, IL-6 induces the passage of stem cells from the G0 phase of the cell cycle to Phase G1, thereby maintaining the cancer stem cell population. Clinically, high IL-6 levels are observed in patients suffering from many cancer types, including breast, colon, or pancreatic cancer, to name only a few [165].

The mechanism, in which IL-6 signaling contributes to tumor progression and metastasis and regulates other cytokines, can be both autocrine and paracrine [166]. It also regulates interactions between cancer and stoma cells, contributing to the maintenance of the complex TME [167]. Lung and hepatocellular cancer cells treated with IL-6 have shown significant increases in mesenchymal markers and reduced E-cadherin levels. On lung cancer tissue samples, it has been noticed that there is a correlation between IL-6 expression and both E-cadherin and vimentin. The counterparts characterized by low IL-6 concurrently exhibited high E-cadherin expression, suggesting the existence of a direct link connecting IL-6 to EMT. Phenotype switching mediated by IL-6 has been, in a detailed study on a molecular level, found to rely on STAT3 activation upon binding of IL-6 with its receptor [168]. In various cancers, different additional EMT-promoting effects of IL-6 have been described. For example, in PC cells, IL-6 mediates the cross-activation of the insulin-like growth factor-1R (IGF-1R) in a paracrine manner, which further stimulates STAT3 [169]. Reports concerning interactions of IL-6 with specific miRNAs, altering the expression of epithelial/mesenchymal markers, are also present in the literature. A crosstalk of IL-6 with TGF-β and Hsp27 with subsequent EMT induction was also reported to occur. Another aspect of IL-6 involvement in EMT is the role in modulating interactions, which include other TME components [170]. IL-6-mediated EMT has also been found enhanced by CCL2, with the following STAT3 phosphorylation creating a positive feedback loop to amplify signaling via upregulating both IL-6 and CCL2 [171].

The other direction of IL-6 and EMT interaction, i.e., if, and how, the acquisition of mesenchymal phenotype by cancer cells promotes the development of inflammatory and immunosuppressive characteristics of TME, is also investigated. In fact, the activated EMT program induces a secretion of soluble factors with inflammatory properties, such as IL-6 and IL-8. Moreover, neoagniogenesis stimulation, manifested by VEGF and angiogenin upregulation, was shown to be induced by cells undergoing mesenchymalization [172].

#### 3.5.5. IL-8

Initially, IL-8 was described as neutrophil-activating and chemotactic factor due to its established role as a proinflammatory cytokine. Stress conditions, such as hypoxic environment, inflammatory signals such as TNFα and IL-1β, or chemotherapeutics additionally enhance its production by cancer cells. Some of these, along with endothelial or immune cells, also express IL-8 receptors (IL-8Rs), formerly known as CXCR1 and CXCR2 (CXC motif chemokine receptor 1 and 2, respectively). The former exhibits specificity towards IL-8, while the other can bind different chemokines, e.g., IL-6, too [145,172,173].

IL-8 is connected with cancer progression on various levels. When secreted by tumor-associated macrophages, cancer cells, or cancer-associated fibroblasts, IL-8 promotes synthesis and the release of other oncogenic growth factors, additionally protecting cancer cells from death by suppressing cytotoxic T lymphocytes [174]. A considerable body of evidence demonstrates that IL-8 can affect a variety of TME components through autocrine or paracrine signaling, which is associated with the recruitment of immune cells of different populations (particularly macrophages and neutrophils) [175]. The local inflammatory response, mediated by IL-8, was accompanied by inducing M2 polarization of tumor-associated macrophages and the following EMT in hepatocellular carcinoma (HCC) cells, as shown by Xiao et al. [176]. Increased expression of MMP-9 was also connected with IL-8 overexpression, contributing to bladder cancer progression [177].

Signaling kinases associated with cancer progression, which are easily activated by IL-8, include MAPK, FAK, and Src, along with NF-kB, PI3K/Akt, and Jak2/STAT3/SNAIL pathways [173]. Due to the presence of IL-8Rs in the endothelium, this chemokine can cause Ras/PI3K pathway activation and NF-κb-mediated VEGF activation [178]. It allows for the presence of IL-8 as a powerful pro-angiogenic factor, promoting the expression of VEGF and its receptors [179].

IL-8/IL-8R axis plays a particularly significant role in EMT mediated by the Brachyury transcription factor, as demonstrated in breast and lung cancer cell lines. An enhanced secretion of this cytokine by Brachyury-overexpressing epithelial cancer cells has been connected with the acquisition and further maintenance of mesenchymal features and increased invasiveness. The exposure of breast epithelial cancer cells to IL-8 promoted a switch into mesenchymal phenotype, manifested by an increase in fibronectin and a decrease in E-cadherin amounts [180]. Studies based on IL-8 inhibition confirmed its importance in SNAIL-induced EMT. Importantly, SNAIL, via direct binding to specific sequences located in the IL-8 gene promoter, is able to activate its transcription and IL-8 expression, creating an autocrine positive loop aggravating EMT progress, as well as in the expression of genes associated with cancer cell stemness such as SOX2, Oct4, and Nanog. In CSCs, the expression of IL-8Rs is elevated in comparison to cells deprived of aldehyde dehydrogenase (ALDH) activity, which is an acclaimed stemness marker, further confirming the link between these [172]. Visciano et al. reported that IL-8 can also activate the SLUG/Akt pathway and induce EMT in thyroid cancer cells [181].

### 3.6. Growth Factors

The process of EMT is under considerable control of growth factors. As mentioned above, TGF-β is of paramount importance in this regard due to its involvement in regulating the expression of other cytokines, the diversity of signaling pathways, and ECM remodeling enabling the penetration of cancer cells through basal membranes [182]. Regarding EMT induction through upregulated SNAIL expression, TGF-β acts in synergy with other growth factors: EGF, FGF, and HGF. In particular, the ways in which FGFs are associated with EMT promotion are described. Since the involvement of the FGF signaling network in cancer progression and contribution to processes such as angiogenesis or inflammation is well established, it was proposed that it might act as an inducer of invasive phenotype development and increase metastatic capacity [183]. In fact, FGFs are able to enhance EMT through their downstream-signaling pathways and effectors–Ras/MAPK, Ras/PI3K/Akt/mTOR, and PLCγ/PKC [184].

An interesting aspect and the additional EMT-triggering mechanism are provided by a unique cross-talk between FGF-2 and TGF-β, which regulates isoform switching of FGF receptors (FGFRs). There are four FGFRs, each built of an intracellular tyrosine kinase domain, a transmembrane domain, and three extracellular immunoglobulin (Ig) loops. Alternative splicing of the FGFR gene products generates diverse isoforms, differing in mediated cellular effects. The splice isoform switching, occurring in the C-terminal region of the IgIII loop, affects ligand binding specificity. TGFβ, via repressing epithelial splicing regulatory proteins (ESRPs), induces FGFR2 switching from the epithelial-Type IIIb isoform to the mesenchymal-like IIIc isoform (FGFR2IIIc). FGF-2 and FGF-4, present in high amounts in stromal tissues, preferentially bind to the latter isoform, and this ligand–receptor interaction tremendously enhances EMT and chemoresistance [185,186]. Several FGFs are able to directly modulate the expression of epithelial or mesenchymal markers. FGF-16 was found to enhance cancer cells’ migratory capability through upregulating MMPs and repressing E-cadherin, while FGF-9 has been reported to induce N-cadherin and VEGF-A in PC cells [183].

Cooperation between EGF and TGF-β was demonstrated on BC cells undergoing EMT. Upon exposure to secreted TGF-β, EGF was released and further activated FAK, leading to cytoskeletal alterations and increasing the motility of cells. Moreover, TGF-β-mediated EMT enhancement correlated with the increased expression of surface receptors for EGF (EGFRs) [187], and EGF/EGFR signaling is known to increase TWIST transcripts and protein level via STAT3 activation, inducing a change into spindle-like mesenchymal morphology [188]. Also, the protease TACE/ADAM17, the activation of which by TGF-β facilitates the release of EGF ligands, is involved in the extracellular mechanism of TGF-β-mediated transactivation of the EGFR pathway [189].

Cho et al. [190], in their study on PC cells, demonstrated that EGF exhibits the capability to activate HIF-1α and subsequently induce expression of TWIST1, causing a phenotypic switch from epithelial to mesenchymal cell types. The authors have provided the explanation that ROS and STAT3 phosphorylation are crucial for this to occur, concluding that EGF promotes PC progression and invasion through a ROS/HIF-1a/TWIST1/N-cadherin pathway. EGF is also involved in integrin/EGFR-ERK/MAPK signaling, promoted by calreticulin, and the EGFR/Ras/ERK pathway [191].

## 4. Epigenetic Regulation of EMT

### 4.1. DNA Methylation and Histone Modifications Affecting EMT-Related Gene Expression

DNA methylation, chromatin remodeling complexes, as well as microRNAs (miRNAs), and long non-coding RNAs (lncRNAs) have been proven to regulate EMT. Other factors such as hypoxia hugely influence the epigenetic EMT regulation. The plasticity and reversibility of epigenetic mechanisms create the potential for control strategies. One of the most important mechanisms inducing EMT is through TGF-β mediation. Studies show that both DNA methylation and changes of different histone marks (H3K27me3, H3K9me3, etc.) take part in EMT induced by TGF-β. DNA methylation has also been linked to mechanisms evading immune surveillance in cancer development [192].

Various epigenetic changes are needed in order for EMT to occur. TWIST recruits the methyltransferase SET8 to promote the methylation of H4K20, a histone associated with the repression of E-cadherin and the activation of N-cadherin promoters, which indirectly increase α-smooth muscle actin (α-SMA) and MMP-9 expression to aid cell invasion.

CAFs and other microenvironmental components release cytokines, which downregulate UHRF1, DNMT1, and DNMT3B and cause rapid, extensive, simultaneous, but also reversible, DNA hypomethylation. Hypomethylation of stem cell genes causes induction of EMT through activation of important transcriptional variants ZEB1 and vimentin, thus increasing cancer stemness [193,194].

One study concludes the DNA methylation of a cadherin gene-DSC3 (desmocollin 3) can predict poor clinical outcomes in PC. Methylation downregulates the DSC3 gene, causing an increased induction of EMT and increased PC cell migration and proliferation [195]. It has been documented that reversible DNA methylation occurs on promoters and gene bodies of TINAGL1, ESYT3, ITGA5, and FKBP10 in human primary breast tumors. These are four genes that undergo promoter-associated DNA methylation and their expression changes in human EMT. TINAGL1 encodes a protein participating in cell adhesion, among its other functions. Integrin-3, encoded by ITGA3, is related to apoptosis resistance and cell migration, while the FKBP10 protein is implicated in cancer cell proliferation, invasion, and migration. It has been proved that in both TINAGL1 and ESYT3 gene promoters, they are unmethylated in epithelial BC, but metastatic cells underwent hypermethylation of both promoters leading to transcriptional silencing. Using the DNA demethylation agent, transcriptional ability can be restored, and expression of the genes achieved. It has been found that increased methylation of ESYT3 corresponds with shorter overall survival (OS) in BC patients. The expression of ITGA5 and FKBP10 has been increased in mesenchymal-like cells in which both genes underwent promoter hypomethylation inflating transcriptional activity [196]. Interestingly, hypermethylation on CpG islands consisting of repeated cytosine and guanine nucleotides inactivates miR-200 promoters in both non-small cell lung cancer and invasive bladder and breast cancer [197]. Another study reports that DNA hypermethylation causes the disruption of oncogene insulators in DNA-binding protein called the CCCTC-binding factor (CTCF), which insulates the chromatin loops and partition of topologically active domains. That allows for greater activation of inappropriate promoter oncogenes through enhancer genes [193].

### 4.2. Chromatin Remodeling Complexes and Their Role in EMT Plasticity

The reprogramming of specific chromatin domains seems to be characteristic of EMT [198]. For EMT to occur, certain epigenetic remodeling is required. We have mentioned some of the effects of methylation and histone modification already. The remodeling of chromatin is also caused by specific chromatin complexes such as Polycomb and Trithorax. These complexes increase cellular plasticity to allow for better access to genes important for EMT. It has been reported that quantity effects specific promoter changes. For example, for H3 lysine 4 trimethylation (H3K4me3), the number of promoters increases by over 20%, while the number of H3K27me3 promoters decreases significantly [199].

Similarly, it has been reported that in cancer genomes, H3K4me3 is more active while H3K27me3 is repressed. Chromatin in cancer cells nuclei shows alterations on multiple levels [193]. In EMT, chromatin remodeling starts by increasing accessibility to the genes. Chromatin becomes overly permissive causing aberrant cell reprogramming. Pioneer TFs open up the chromatin to other TFs, which is when histone methyltransfeeases and factors such as EZH2 (Enhancer of Zeste Homolog 2), p300 (histone acetylation enzymes), ubiquitinating enzymes, and others affect the chromatin. Due to changes in chromatin plasticity, EMT is enforced by alternative splicing, as well as post-translational modifications [200]. FGFR2, CD44, catenin (cadherin-associated protein), delta 1 (CTNND1, or p120- catenin), and the Enabled Homolog (ENAH) are all genes that through regulation by ESRP1 and ESRP2 are subjected to alternative splicing during EMT. The serine/arginine-rich splicing factor 1 protein (SRSF1, or SF2/ASF) is also involved in EMT [201]. In EMT induced by TGF-β global chromatin, changes have been reported to influence regulation of EMT-TFs and EMT markers. The removal of a histone variant H2A.Z and chromatin modifiers, such as UTX, Rad21, PRMT5, RbBP5, and others, have been identified to have an impact on EMT [192]. We can name HDAC1, HDAC2, and HDAC6, among others. The recruitment of these complexes to promoters could play a role in SNAIL and slug e-cadherin repression in EMT. These complexes catalyze histone acetylation in EMT in cancers [201].

Histone changes allow for chromatin alterations, which then enable EMT. It has been reported that during EMT, in large, organized heterochromatin K9 (LOCKs), the heterochromatic mark histone 3 (H3)-lysine 9 dimethylation (H3K9me2) is reduced, while the euchromatic marks H3K4me3 and H3-lysine 36 trimethylation (H3K36me3) are increased in euchromatin [201]. The increase in H3K4me3 and H3K36me3, as well as loss of H3K9me2 during TGF-β-activated EMT, was reported to be, in part, dependent on lysine demethylase-1 (Lsd1). The same study also suggests that the influence of Lsd1 can be diminished by pargyline (a monoamine oxidase inhibitor that blocks the ability of this enzyme to demethylate H3K9me2 within chromatin) [198].

During EMT, PRC2 subunits EZH2 and Suppressor of Zeste 12 (SUZ12) catalyze the trimethylation of K27 on histone H3 (H3K27me3) in the nucleosomes surrounding promoters, which results in transcriptional repression. Afterwards, PRC1 is recruited. Its subunits CBX2, CBX4, and CBX8 recognize and bind previously methylated histones. Another subunit of PRC1- BMI1 seems to play an important role in the formation of more mesenchymal, CSC-like cells as its upregulation is well reported in invasive tumor phenotypes [197]. EMT has also been proven to be induced by the subunit of SWItch/Sucrose Non-Fermentable (SWI/SNF) ATP-dependent chromatin remodeling complex, protein BRG1, possessing the ATPase activity, which interacts with ZEB1. It suppresses E-cadherin independently of C-terminal binding protein (CtBP) through remodeling the local structure of chromatin around the gene [202]. One study reports that in BC, the activity of methyl CpG-binding domain protein-3 (Mbd3), a crucial scaffold protein of the nucleosome remodeling and deacetylase complex (NuRD), along with histone deacetylases (HDACs) and Tet2 hydroxylase, preserve the mesenchymal state. Interference in their function leads to MET. Therefore, an inhibition of these factors could prevent the progression of EMT and metastasis [203].

It has also been demonstrated that Grainyhead-like 2 (GRHL2) acts as an EMT suppressor in ovarian cancer. An increase in methylation found on CpG sites of promoter genes and nucleosome remodeling is reported to be an effect of GRHL2 knockdown. An increase of H3K27me3 is shown, while the levels of H3K4me3 and histone H3 acetylated at lysine 27 (H3K27ac) are reduced. This change is characteristic in progressive EMT. Therefore, it can be concluded that GRHL2 and other factors can regulate the availability of genes for epigenetic changes. This study suggests that in the case of low chromatin accessibility, which allows for epigenetic changes, EMT cannot occur [204].

### 4.3. Non-Coding RNAs (miRNAs, lncRNAs) as Regulators of EMT Signaling

The competing endogenous RNAs (ceRNAs) create a regulatory network in which the expression of both coding and non-coding DNA is altered. Both miRNAs and lncRNAs influence this network. Some of the single aberrantly expressed ceRNA occur in the double-negative feedback loop in EMT. The ultimate outcome of the regulation could be EMT and progression of cancer. It has been reported that ceRNAs tend to compete with the miRNA-200 family, which is the one having the most impact on EMT [205].

MiRNAs have been proven to be important regulators of EMT signaling, with an expression ofmiRNA-200 family members most altered during EMT. It has also been reported that these miRNAs have high diagnostic accuracy and can be used as biomarkers for BC prognosis [206]. The MiRNA-200 family comprises, among others, miRNA200a, miRNA-141, miRNA-200b, miRNA-200c, and miRNA-429, which are the transcriptional targets of ZEB1 and ZEB2 with a double-negative feedback loop established between them. miRNA-200 family downregulation and ZEB1 upregulation support EMT promotion [205]. There is evidence that miRNA-203 could be a new diagnostic and therapeutic target for the treatment of head and neck squamous cell carcinoma (HNSCC) due to differences in its expression levels between invasive and non-invasive subclones of this cancer. It has been suggested that miRNA-203 suppresses EMT induction through targeting NUAK1 (gene involved in invasion and EMT induction in HNSCC). An increased expression of miRNA-203 was found to suppress EMT in HNSCC, causing SNAIL2 downregulation. The hypermethylation of miRNA-203 leads to a loss of its suppressive function, which is observed in many cancer cells, including HNSCC [207].

It has been reported that in PC, miRNA-203 is downregulated. It has been found to inhibit EMT in PC, as well as in ovarian cancer cell lines, by downregulating BIRC5 and attenuating the TGF-β signaling [208]. In non-small cell lung cancer, invasive bladder cancer, and BC, hypermethylation on CpG islands inactivates the miR-200 promoter. A demethylating agent or a miR-200 analog could promote epithelial redifferentiation, which could restore chemosensitivity in resistant cancers [197]. One study focused on the influence of different miRNA types on EMT in PC. In the tabular summary, it demonstrates that miRNA-3622a, miRNA-466, miRNA-543, miRNA-132, miRNA-212, miRNA-143-3p, miRNA503, miRNA-409-3p, miRNA-301a, miRNA-210-3p, miRNA-573, miRNA-802, and miRNA-410-3p, as well as the already-mentioned miRNA-203, all influence EMT in cancer. The results present which other functions these miRNAs have in cancer, but, most importantly for our inquiry, it shows which possible mechanisms deregulate these miRNAs. Some miRNAs promote EMT (miRNA -802, -543, -503, -466, -410 -3p, -409-5p, -409-3p, -301a, -210-3p), while others inhibit this process (miRNA -3622a, -573, -212, -203, -141-3p, -132). An overexpressed miRNA-210-3p and downregulated miRNA-141-3p are reported to have great significance in promoting metastasis formation. It has also been suggested that miRNA-543 should be considered as a predictor of the risk of metastasis, which is based on vast evidence that proves it to be involved in EMT initiation through the Raf kinase inhibitor protein-RKIP. In BC, miRNA-410-3p decreases migration and the invasion of rhabdomyosarcoma cells by inhibiting EMT [208].

Other miRNAs such as miRNA -300, -382, -494, -495, -539, and -544, as well as the already-mentioned miRNA-543, have been found to promote EMT by repressing signaling networks through the hypermethylation of upstream CpG islands in human ductal carcinomas. Those signaling networks include TWIST1, BMI1, and ZEB1/2, but also the miRNA-200 family, whose importance we have already discussed. It is, therefore, another evidence that miRNA expression levels and methylation status could be used for diagnosis and as therapeutic targets [209].

Some studies suggest that sensitizing CSCs to traditional therapies can be achieved through the use of epithelial-scpecific microRNAs, such as miR-200, miR-34 (miRNA interacting with SNAIL), and let-7 [197,210]. Let-7g has been reported to influence HCC. It downregulates the k-Ras/ERK1/2 pathway, therefore suppressing EMT. Results show that a low expression of let-7g is associated with poorer survival outcomes with HCC [211].

In human gastric cancer, a decreased expression of miRNA-1228, in comparison to healthy tissue, has been reported. Results suggest that NF-kB, which plays an important role in the progression of EMT in multiple cancers, could be downregulated through a process mediated by miRNA-1228. That, and perhaps targeting casein kinase 2-alpha 2 (CK2A2) expression, could explain why the restoration of miRNA-1228 in gastric cancer tissue suppresses EMT [212]. It has been reported that hypoxia activates miR25/93 to repress the cGAS-STING pathway and mediates immunosuppression in tumors [192].

Long non-coding RNAs (lncRNAs) are dysregulated in various cancers and can function as tumor suppressors, as well as oncogenesis drivers. For example, the progression of breast cancer is supported by H19 and TP73, metastasis-associated lung adenocarcinoma transcript 1 (MALAT1) aggravates renal cancer, non-small cell lung cancer, and thyroid carcinoma, ZEB1-AS1 is involved in intrahepatic cholangiocarcinoma, SNHG15 in papillary thyroid carcinoma, HCC, colorectal cancer, osteosarcoma, and nasopharyngeal carcinoma, and *Bacteroides fragilis* lncRNA1 (BFAL1) is linked to bacteria-induced carcinogenesis. The mechanism of lncRNA action involves miRNA sponging (i.e., specific inhibition of miRNAs complementary to particular lncRNA), thereby regulating the expression and activity of certain miRNAs implicated in the EMT phenomenon. Specifically, sequestering miRNA141 and miR-200 families by some lncRNAs has a proven effect on EMT [205]. lncRNA SPRY4-IT1 (intronic transcript 1) regulates EMT in NSCLC (non-small-cell lung cancer). Reportedly, it mediates the inhibition of cell migration, invasion, and metastasis, possibly through its inhibition of EMT. The evidence shows that the lncRNA SPRY4-IT1 is epigenetically silenced in NSCLC through transcriptional repression mediated by the Polycomb group protein enhancer of zeste homolog 2 (EZH2). Therefore, it can be used as a prognostic factor for poor survival rates and a higher risk of metastasis [213]. Upon its enforced expression, another IncRNA HOTAIR promotes BC metastasis by interacting with PRC2 and H3K27me3 [197].

Circular RNA was also reported to influence EMT. In contrast to hsa_circ_0008305, which inhibits EMT, most circRNA promote EMT through miRNA sponging. CircRNA like: hsa_circ_0057481, hsa_circ_001783, and circ_101368 affect miRNA-200 family, but circ_GNB1 sponges miRNA-141. Similarly, pseudogene-derived RNA molecules have been found to promote the initiation of EMT by sponging mRNAs [205].

## 5. Cellular Plasticity and EMT

### 5.1. Hybrid Epithelial/Mesenchymal States and Their Relevance in Cancer Progression

Nowadays, EMT is no longer considered a binary process, but EMT-undergoing cells present a spectrum of transition states between epithelial and mesenchymal phenotypes. According to the conventional EMT paradigm, cancer cells detaching from primary tumors present sole mesenchymal (M) markers, while before conversion and after settling at the metastatic site, they express markers associated with the epithelial (E) type. In fact, E/M or M/E interconversion involves the co-existence of cells presenting various epithelial and mesenchymal features, which remain in plastic, transient forms. This phenomenon is referred to as hybrid EMT, also known as partial or intermediate EMT. Such plasticity aids disseminating cells to endure stress conditions and adopt to the challenging microenvironment [214,215].

Evidence for gradual transformation of E-like cells has been collected throughout studies on clinical samples obtained from cancer patients. CTCs from patients with advanced cancers have been found to express both types of markers and present collective migration patterns, which occur when cells migrate in clusters. The leading cell exhibits a hybrid phenotype with visible mesenchymal traits and high actin-dependent mobility, while cells located in the cluster center retain polarity and intercellular junctions [216]. Hybrid E/M states have been noticed to occur in primary tumors as well, as reported for breast, lung, and PC, and in xenograft models [217]. Between the two extremes of complete epithelial or complete mesenchymal phenotypes, multiple intermediate cell states may exist, and their number is a debatable issue. For instance, upon EMT induction through TGF-β in MCF10A breast tissue cells, one intermediate state was found, while in the case of BC patient-derived CTCs, three different hybrid phenotypes existed [218].

E/M plasticity (EMP), defined as the ability of cells to switch between various hybrid E/M states, is strictly connected with cancer progression on several levels, including migration, stemness development, metastases formation, and chemotherapy resistance [219]. Intermediate E/M states exhibit strong tumor-initiating potency and cellular plasticity [220]. It is indicated that cells bearing hybrid E/M phenotypes possess higher metastatic activity than mesenchymal-like cancer cells. As demonstrated, the concurrent expression of vimentin and Cytokeratins 8 and 18 has a stronger influence on invasiveness, enhancing metastasis [214]. The collective migration of disseminating cells, relying on partial EMT as aforementioned, is suspected to be the predominant strategy of breast, lung, colorectal, and pancreatic cancer metastasis, based on the 3D tumor model study. Single-cell migration was calculated to comprise far less than 1% [221]. Moreover, collective invasion also greatly contributes to the acquisition of CSC properties, as well as the opposite [222]. Hybrid E/M BC cells co-express aldehyde dehydrogenase (ALDH) and CD44, the established CSC markers, and are characterized by a high mammospheres-forming capacity [223]. Bierie et al. distinguished among TNBC-representing cells an integrin-β4-expressing subpopulation, enriched in CSC features and representing an intermediate EMT state [224]. Partial-EMT+ breast CSCs were shown by Papadaki et al. to survive first-line chemotherapy in abundance among four subpopulations differently expressing E and M markers [225]. Overall, a partial E/M state is seen as more flexible and exhibits a higher potential to develop stem-like features, increasing tumor heterogeneity.

### 5.2. Phenotypic Switching between Epithelial and Mesenchymal State

The maintenance of E/M plasticity is, to a large extent, mediated by the surrounding microenvironment, with a particular emphasis on stromal cells, including CAFs and TAMs [226]. TME conveys EMP-inducing stimuli, recognized as mechanical stress, immune responses, ECM remodeling, hypoxia and HIF1α activation, and exposure to chemotherapeutics. The stiffening of ECM activates integrin signaling and PI3K, MAPK/ERK, and Rho/ROCK pathways, enhancing the cancer cells’ migratory capability [227]. The establishment of intermediate E/M states has been found mediated by the interactions between EMT-TFs and microRNAs, with the best-known example to date being miR-200/ZEB [228]. ZEB factors are well-established transcription repressors, downregulating multiple epithelial genes, including E-cadherin, while the miR-200 family suppresses EMT and triggers epithelial differentiation. The miR-200 overexpression negatively influences Zeb1 expression, and vice versa. ZEB1 can downregulate miR-200, leading to a double-negative feedback-loop generation. Therefore, the ZEB/miR-200 loop controls many intracellular decisions, including functional states of cells, such as stemness, cell cycle arrest, survival, or senescence; the interrelated differential expression of ZEB factors and miR-200s, in large part, dictates EMP [229].

Prolonged exposure to TGF-β has been connected to the hybrid E/M states due to its role in the modulation of two EMT-TFs: SNAIL and PRRX1. The former, with a role in epithelial gene downregulation, is repressed, while the latter promotes the expression of mesenchymal genes and is activated through a feed-forward loop. However, rapid stimulus from TGF-β induces SNAIL and represses PRRX1, leading to hybrid E/M state acquisition [230].

The development and application of mathematic models, in order to identify factors stabilizing the transient phenotypes, allowed us to pinpoint NRF2, NUMB, OVOL2, and GRHL2 as such phenotypic stability factors [231]. The elucidation of cellular mechanisms regulating hybrid E/M states is much more complicated than in the case of binary E or M fates due to their great diversity. Also, the epigenetic landscape is complex and not yet comprehensively understood, with a need for further studies [217].

### 5.3. Plasticity-Driven Drug Resistance and Implications for Therapy

Despite the constant increase in various cancer-treatment options, the acquisition of resistance is still a major challenge and reason for therapy failures. It concerns not only chemotherapy but also immunotherapy and targeted therapies with the use of monoclonal antibodies [232]. Although intermediate E/M phenotypes have been clinically associated with resistance to anticancer therapy, the molecular bases remain largely elusive [215]. It is mainly attributed to the generation of CSCs, which exhibit enhanced drug efflux. They often remain in dormancy, protected from treatment-induced death [215]. Due to high transdifferentiation potential, CSCs provide lineage plasticity, enabling an adaptation to changing extracellular stimuli and stressors [227]. Moreover, there is an evident link between chemoresistance and tumor heterogeneity driven by phenotypic plasticity [233].

Considering conventional chemotherapy, EMP has been reportedly implicated in advanced PC resistance to docetaxel and combined cabazitaxel/antiandrogen treatment. Docetaxel failure was mediated by the reduced expression of miR-200 family members involved in the EMP regulatory loop, as demonstrated by Puhr et al. [234]. One of the mechanisms involved in the resistance of malignant melanoma cells to B-Raf protein kinase inhibitors (BRAFi) was shown to be ZEB1 reprogramming towards phenotypic switching from E to M and the upregulation of stemness markers [235]. EMP in melanoma cells, through activation of the unfolded protein response (UPR), mediates both BRAF and MEK inhibitors (MAPKi), as well as anti-PD-1 therapy resistance [236,237].

Intermediate E/M states are suspected to confer therapy resistance in triple-negative BC cells to a greater extent than mesenchymal cells with the completed EMT program, but the exact mechanistic pathways are currently only studied [233]. The connection between hybrid E/M states and chemoresistance was well demonstrated on breast CTCs. Relying on RNA in situ hybridization and RNA sequencing in order to analyze the expression of epithelial and mesenchymal markers, Yu et al. [238] have proven that an abundance of biphenotypic cells was associated with increased resistance to combined chemotherapy and targeted therapy, based on various agents. However, the elucidation of EMP influence on therapy resistance in the clinical context is a complicated issue with many factors coming into play, such as multidrug regimens, where distinguishing the relevance of cell plasticity to the development of resistance to the specific agent can be impossible.

The identification of different cell subpopulations in tumors and transition E/M states could be utilized in adjusting treatment aiming towards specific phenotypes. There are some data available pointing out that EMP has an important contribution to immune evasion. Broadening this knowledge might lead to the potential application of phenotypic assessments to find predictive factors for the choice of the most efficient immune therapy. Moreover, the phenomenon of EMP can be made use of to favor drug-induced reversion to epithelial phenotype or transdifferentiation into post-replicative states, which would reverse drug resistance and sensitize cells to applied chemotherapeutics, as well as reduce stemness [233].

## 6. Experimental Models for Studying EMT

### 6.1. In Vitro Models (Cell Culture, Organoids) to Investigate EMT Mechanisms

Many in vitro models have been developed for studying EMT mechanisms. In 2010, a study proposed a measure of spindle phenotype-spindle index, which is the ratio of maximum length to the maximum width (measured normal to the length axis) of each cell. A novel chemoattractant assay has been proposed for measuring the area covered by cells in migration from a fixed coverslip (10 mm) towards a supplemented agarose (with RPMI supplemented with FCS concentration above 50%). There is a 1 mm gap established between agarose and central coverslip, as it was found to be optimal for cell migration. Afterwards, the central portion of agarose is removed, remains are washed twice in SdM, and the wells can be scanned using light and phase-contrast microscopy [239].

A 2011 study proposed a 3D in vitro invasion model that has been developed for cell lines with EMT features in BC. The model mimics the process of cells with an invasive phenotype, breaching the basement membrane (BM) into the stromal collagen, and it resembles aspects of EMT in vivo. The basement membrane is mimicked by laminin-rich BM-like Matrigel, in which histologically normal breast epithelial cells are first embedded. Afterwards, everything is embedded into collagen. Models used in older studies used a pure collagen environment or non-physiological methylcellulose. Others used BM-containing models or peritoneal BM, which are inflexible, difficult to scale up, and often produce very low yields. This model allows us to study cells that are contained in BM, invading across BM, and invading more distally into surrounding collagen. The movement of cells can be followed by light microscopy [240].

A newer study (2016) reports that 3D models using Matrigel, as well as extracellular matrix-derived protein gels, often present results that are difficult to compare between different laboratories and compartments. A different 3D model has been suggested in which the inducible epithelial cell line (EpH4)12,13 and a bioengineered ECM-like matrix with independently tunable properties are combined. The proposed matrix is an optimized soft alginate hydrogel functionalized with cell-adhesive RGD peptides with supportive tissue. It offers the ability to promote integrin-mediated cell adhesion and cell-matrix crosstalk, as well as easily altered concentration and molecular weight. This model allows for TGF-β1–induced EMT and its reversion, as well as the generation of cells with epithelial-like, mesenchymal-like, and intermediate phenotypes. The adequate microenvironment proposed by this model can mimic native tissues, like mammary tissue [241].

Another study, conducted in 2023, uses BM-typical globular collagen Type IV and stroma-typical collagen Type I coatings as provisional 2D matrices for an in vitro investigation of EMT phenotypes in BC. Collagen Type IV is used as BM. Collagen Type I is used as the main extracellular compound, which is separated from epithelium by BM in the healthy tissue. Tumor progression disrupts the separation and creates leakage between epithelium and extracellular compounds [242].

There have been significant differences, which show a superiority of 3D cell cultures over 2D cultures grown in the monolayer. One study reports that 3D HNSCC cell cultures effectively mimic the in vivo behavior of neoplastic tumor cells. The 3D model also shows decreased sensitivity to cisplatin and cetuximab (anti-EGFR) treatment [243]. One study from 2016 has also reported on the benefits of a 3D model organoid model instead of 2D. The article reports that HCC cells in porous alginate scaffolds can generate organoid-like spheroids, which mimic, in many ways, HCC cells in vivo. For the model to work, an activation of the Wnt/β-catenin-signaling pathway is required so that the HCC cells maintain stemness crucial for spheroid formation. The proposed model shows increased sensitivity to TGF-β-induced EMT, as well as a resistance to chemotherapy in comparison to 2D models [244].

In vitro tumor spheroids are small clusters of cancer cells formed through 3D cell-culture techniques. They come in two types: homotypic (made up of only cancer cells) and heterotypic (including both tumor and stromal cells). These spheroids can be created using scaffold-free or scaffold-based methods, each with its own pros and cons. Various techniques, such as spinner flasks, hanging drop, magnetic levitation, ultra-low attachment plates, microwells, macroscopic 3D matrix, microgels, and 3D bioprinting, are utilized for their formation [245].

In vitro models of EMT frequently involve co-culturing cancer cells with CAFs. Harner-Foreman et al. established a co-culture system where HT-29 tumor spheroids (TSs) were placed in proximity to CCD-18 fibroblasts. After co-culturing with TSs, CCD-18 co-fibroblasts showed signs of activation, increased proliferative activity, and enhanced cell migration. This co-culture system mirrors the EMT state observed at the invasive edge of tumors during early metastasis in vivo [246].

Organoids, 3D structures from patient samples, surpass tumor spheroids in disease modeling and drug screening. They mimic in vivo tumors well and are used for various cancers (lung, breast, pancreatic, colorectal, ovarian, prostate, and stomach). Matrigel, a traditional matrix, has limitations, leading to the exploration of alternative biomimetic hydrogels such as enzymatically crosslinked gelatin, synthetic PEG-based hydrogels, nanofibrillar hydrogels, and hybrid collagen-hyaluronic acid hydrogels, among others. These alternative matrices offer advantages such as tunable stiffness, composition, and reduced immunogenicity [245].

In a study from 2014, a 3D organoid model was created in a rotating wall vessel bioreactor based on liver cells. The organoids were inoculated with colon cell carcinoma, creating liver–tumor organoids, which allow for in vitro modelling of liver metastasis. The model allowed for observing phenotypic changes and EMT [247].

Organoid models have also been used in a reproducible scaffold-free 3D model to study neoplastic progression and EMT in BC. It has been suggested that the scaffold-free model has many advantages, amongst which are high consistency and reproducibility. It also allows for stimulating the organoid in many ways without concern for matrix binding and measuring cellular Collage I production with no interference from the exogenous collagen. The study reports that the hanging drop-system 3D organoid model has advantages over both u-bottom spheroid formation, as well as conventional scaffold-based Matrigel culture. It allows for the expansion of the cell populations with normal and neoplastic phenotypes and is very effective as it is supportive of organoids by providing more oxygen and enhancing autocrine effects relative to the well plates by using smaller media volumes [248].

Additional research reports that the use of organoids were derived from circulating tumor cells, which exhibit a hybrid EMT state and show several features similar to colorectal CTCs. Therefore, they can be used to investigate the features of metastatic CRC cells. Potential applications include identifying new prognostic biomarkers and devising new potential strategies for metastasis prevention [249]. Organoids-on-a-Chip platforms provide a dynamic nutrient supply and mimic physiological flow, improving drug-testing accuracy. Organoids-on-a-Chip platforms combine microfluidic technology and organoids. These can provide a dynamic supply of nutrients in a physiological flow. These platforms have been used for drug-sensitivity testing and mimicking pathological flow environments, such as vascularized tumor organoids-on-a-chip, which replicate in vivo drug responses and provide the potential for assessing patient-specific responses to chemotherapy. Miniaturized organoid versions enable high-throughput drug screening. Challenges remain, but ongoing clinical trials show promise for organoids in drug screening and cancer studies [245].

### 6.2. In Vivo Models for Studying EMT in Tumor Progression and Metastasis

Here, we have described a few in vivo models on mice, which have been developed for studying tumor progression and metastasis. One study reports the use of female SCID mice, which were implanted subcutaneously in the flank with H358 TET-ON, preTre2- SNAIL, pTRE2-Zeb1, or pTRE2-aTGF-β cells, which were suspended in normal growth medium in a 1:1 ratio with Matrigel. Mice were left for 7 days to let the tumor grow. Afterwards, mice were sorted so that only mice with tumors with a mean volume of about 250 mm^3^ prior to induction of transgenes were included. Chosen mice were administered 0,5mg/mL of doxycycline in drinking water (1 week for ZEB1 and SNAIL, 2 weeks for TGF-β). Following that, tumors were measured on the 7th, 14th, and 21st day post-implantation and were harvested on the 21st day. This shows an example of an in vivo mouse model for studying EMT [250].

Santamaria et al. created a genetic mouse model for evaluating the relevance of EMT in the metastatic process. They chose three steps in the metastatic process, invasion, dissemination, and distant metastasis, for targeting with different EMT-TFs (TWIST1, SNAIL1, ZEB1) based on their knock-in or knock-out. Depending on the tumor stage, different EMT-TFs are activated or inactivated. The occurrence of EMT is shown by a color code of fluorescent reporters driven from promoters in different cancer models. Various cancer models have been used (SCC, BC, pancreas) [251].

Another study performed by Bonnomet et al. used mice in order to investigate EMT in circulating tumor cells and metastases of BC. Transplantable human BC cells inoculated with Matrigel have been implanted subcutaneously into 6-week-old immunodeficient SCID mice. Tumor growth was measured every 10–15 days. In all cases after killing, tumors and organs were collected and analyzed immunohistochemically or microscopically through laser capture; densitometry was also performed [252].

### 6.3. Advances in Imaging and Single-Cell Technologies for Dissecting EMT Dynamics

In 2018, it was suggested that the most important challenge for EMT research is to be able to monitor all states of EMT closely from the primary tumor to the metastatic site in improved animal models [253]. Advancement in EMT imaging is needed. Short-term assessments (hours) can be achieved with many techniques, such as intravital microscopy, but vascular changes happen over days or weeks. One study reports on the development of the novel fluorescent EMT reporter. It has been used within the liver-on-a-chip platform, a method of in vitro modeling, which has been already mentioned in this article. This platform offered an optimal microenvironment for studying and imaging vascular changes in EMT in 2D and 3D cultures. The application of EMT reporters such as CNN1-Rep allows for high-resolution imaging of EMT behavior [254].

Single-cell technologies can also be used for investigating EMT. Single-cell sequencing studies have revealed that EMT is not a binary process but a heterogeneous and dynamic disposition with intermediary or partial EMT states. Other discoveries have been achieved using single-cell sequencing as well [255]. One study used single-cell RNA-sequencing analysis to reveal CMS subtype-specific patterns of epithelial-mesenchymal heterogeneity [256]. Single-cell RNA sequencing used to investigate lymph node metastasis in HNSCC identified a subpopulation of pre-metastatic cells driven by actionable pathways [257].

Computational frameworks can be developed to predict the timing and distribution of EMT from single-cell sequencing data using single-cell RNAseq. Najaf et al. modeled EMT population dynamics with a three-state continuous-time Markov chain and considered phenotypic stability factors that augment transitions into the hybrid state, as well as symmetric stochasticity of the underlying biological mechanisms (intrinsic noise), which tunes the temporal evolution of EMT trajectories. Although the framework had success in the performed study, the researchers also note that future investigation is required to assess the applicability of the method [258].

### 6.4. Predicting Critical Transitions in EMT: From Biomarkers to Advanced Mathematical Models

EMT is characterized by critical transitions, marked by tipping points. Tipping points such as saddle-node transitions and the emergence of destabilized, intermediate, unstable cell-state signals for imminent, critical transitions, which are hard to predict and are very significant for understanding the dynamics of the EMT process. Methods such as DNB (Dynamic Network Biomarker) are being developed to predict tipping points [259]. However, studies suggest that instead of focusing only on molecular parameters, a systematic examination of the whole microenvironment is needed for sufficient EMT prediction. Many parameters are being proposed, such as motility, invasiveness, and other specific molecular/histological markers [260]. New advanced methods utilize single-RNA sequencing in order to build multivariate computational models capable of identifying the tipping points and characterising Gene Regulatory Networks (GRNs) associated with transitions [261].

Landscape Theory and Potential Barrier Height are concepts that enable the creation of comprehensive mathematical models for EMT. Landscape Theory is used to visualize complex EMT processes with many intermediate states through the mapping of EMT onto a metaphorical landscape in which stable cell states are influenced by many factors and undergo transitions on different paths of the landscape. Potential Barrier Height refers to the energy that must be overcome for a cell to transition between states. It is an accurate indicator of critical transitions. Landscape Theory combined with Potential Barrier Height enables the prediction of Early-Warning Signals (EWSs) and critical tipping points in EMT [262]. EWSs such as variance, autocorrelation, and conditional heteroskedasticity are used to signal critical transitions between epithelial, hybrid, and mesenchymal phenotypes in EMT. Monitoring key indicators and dynamic biomarkers, which fluctuate strongly near tipping points, can predict EWSs [263]. By integrating these approaches, researchers are moving closer to predicting and potentially intervening in the critical transitions that drive cancer metastasis.

## 7. Clinical Implications of EMT

### 7.1. Prognostic Significance of EMT Markers in Cancer Patients

As described in the previous sections, numerous signaling pathways and transcription factors are involved in EMT, including the TGF-β pathway, Wnt–β-catenin, and factors like SNAIL, Twist, and ZEB1, among others [253]. The impact of EMT can vary depending on the type of cancer [264]. EMT-TFs play an important role in all stages of cancer progression, from initiation to primary tumor growth, invasion, spread, metastasis, colonization, and therapy resistance [253].

The discovery of predictive biomarkers allows for the combination of companion diagnostics with specific treatments, while prognostic indicators enable the intensity of treatment to be adjusted based on the risk of tumor recurrence or progression [265]. The prognostic value of EMT markers in BC patients has garnered considerable attention in recent research. EMT markers, particularly those associated with CTCs, have emerged as crucial indicators of patient outcomes. Meta-analyses indicated that patients with higher levels of EMT-CTCs had worse OS and progression-free survival (PFS) among BC patients [266,267]. Additionally, the impact was even more significant among patients with cancer in a primary stage than metastasis [266,268]. Certain genes and microRNAs, like ALDH1A1, SFRP1, COL17A1, TP63, miR-21, and miR-200c, have prognostic significance in BC. They provide valuable insights into disease progression and potential targets for therapy [269]. For instance, ALDH1A1, a marker associated with CSCs and EMT, is implicated in chemoresistance and poor prognosis [270].

Depending on the tumor stage, TGFβ in breast tumors exhibits both tumor-suppressive and promoting effects. TGFβ inhibits cell proliferation and stimulates differentiation in normal cells, suppressing tumorigenesis at early stages. Studies showed that in advanced stages, TGFβ usually functions as a tumor promoter, e.g., by stimulating EMT, leading to enhanced invasiveness and metastasis [271,272]. Furthermore, alterations in cell-adhesion molecules like E-cadherin and CD44 are indicative of EMT and metastatic potential in BC. The shift from E-cadherin to N-cadherin expression signals a transition to a more invasive phenotype, promoting tumor metastasis. Additionally, aggressive tumor behavior and EMT progression are correlated with an overexpression of CD44, particularly its standard isoform (CD44s). Additional important factors associated with EMT-mediated BC metastasis include β-catenin and discoidin domain receptor 2 (DDR2). DDR2, a collagen-specific receptor, interacts with extracellular matrix components, facilitating cell invasion and metastasis [18].

In pancreatic cancer, EMT is associated with metastasis and gemcitabine resistance, which depends on the expression of ZEB1 and vimentin [273]. Arumugam et al. found that there is an inverse relationship between the levels of E-cadherin and ZEB1, a transcriptional suppressor of E-cadherin, which closely correlates with resistance to gemcitabine, 5-fluorouracil, and cisplatin [273]. A reversal of the EMT phenotype has been shown to restore drug sensitivity [274]. MUC1, a transmembrane mucin glycoprotein, has been linked to the most aggressive types of pancreatic cancer [275]. According to Roy et al., the overexpression of MUC1 in pancreatic cancer cells induces an EMT process, enhancing invasiveness and metastasis. As a result, MUC1+ cells gained mesenchymal markers such as SLUG, SNAIL, and vimentin while losing E-cadherin expression [275,276].

In HCC, EMT-mediated progression is influenced by tumor-associated inflammatory cells. The significant malignancy of HCC, among others, is its insensitivity to therapeutic agents. An association was found between HCC patients with soRafenib resistance with the degree of EMT of liver cancer cells. This is associated with the downregulation of TGF-β- and PI3K/AKT-signaling pathways by miR-216a/217. It is supposed that the reduction of miR-216a/217 expression may block TGF-β pathway activation to overcome the resistance generated by EMT72 [277,278,279]. Additionally, S100A9 has been identified as a diagnostic biomarker for various cancers, including HCC, with its elevated expression correlating with HBV-positive HCC tissues and promoting growth and metastasis in vitro and in vivo [277,280]. Elevated levels of S100A9 expression detected in serum indicate an unfavorable prognosis for patients after radical HCC resection [281].

Studies have shown that TMEM45A is associated with chemotherapy resistance in human BC and HCC cells, particularly under hypoxic conditions. Additionally, it promotes the growth and invasion of human ovarian cancer and brain cancer cells [282,283,284]. In particular, studies have shown that the inhibition of the TMEM45A gene effectively inhibits multidrug resistance and EMT by disrupting the TGF-β-signaling pathway in human colorectal cancer cells. These findings suggest that TMEM45A has potential as a biomarker [282,285].

Due to the broad concept of EMT, different substances acting on various uptake points are being studied in cancer therapy. By determining the EMT markers, we may be able to unveil the prognostic effects of EMT on tumor progression and metastasis. This also provides an opportunity to develop diagnostic tools and choose appropriate treatment strategies for target patient groups.

### 7.2. Targeting EMT for Therapeutic Intervention

As our understanding of the intricate mechanisms regulating the EMT program grows deeper, there is an undeniable trend towards discovering novel agents that target this program. As mentioned earlier, it is becoming increasingly clear that during EMT, cells go into a less differentiated state, which increases their resistance to therapeutic attacks. Depending on the sources, different strategies are highlighted that seem promising in controlling EMT-mediated tumorigenesis. Preventing the initiation of EMT activation, selective elimination of mesenchymal tumor cells, and reversing the EMT process through induction of a MET program are mentioned [265,286].

Combating EMT by intervening in TGF-β signaling is one well-known strategy. Galunisertib, a small TGF-βR1 inhibitor, has shown promise in treating various cancers by inhibiting the EMT process. Clinical trials have confirmed its efficacy, such as in combination therapy with gemcitabine for advanced pancreatic cancer and with sorafenib for hepatocellular carcinoma. Other small inhibitors targeting TGFβR1, such as EW-7195, LY580276, and SD-208, have also shown anti-tumor effects. In addition, antisense therapies that target TGFβ2 mRNA have shown comparable effects in clinical trials [265,286].

Activation of the HGF axis (also known as Met) is another known mechanism of EMT induction. Often, overactivation of this signaling pathway results from point mutations or amplification of the HGFR gene, leading to increased motility and resistance of EMT-induced cells to anticancer drugs [286,287]. Therefore, significant research is underway on HGF-HGFR-signaling inhibitors, including c-Met inhibitors such as tepotinib and capmatinib. The results of clinical trials are promising, especially for metastatic colon cancer and non-small cell lung cancer with EGFR mutations [288,289].

In addition, potential therapeutic agents targeting EMT induction include COX-2 inhibitors such as celecoxib and AXL inhibitors such as cabozantinib. Phase I clinical trials combining cabozantinib with atezolizumab for the treatment of advanced solid tumors have shown promising results, particularly in the treatment of advanced renal cell carcinoma [286,290].

In colorectal cancer cell lines, research has shown that celecoxib, a selective COX-2 inhibitor, can counteract EMT-related alterations. This includes preventing changes in β-catenin intracellular localization, as well as in vimentin and E-cadherin concentrations induced by hypoxia or EGF [291].

Instead of preventing EMT induction, another promising strategy involves targeting EMT-induced mesenchymal-like cancer cells by inhibiting the functions of EMT-specific markers. For example, ADH-1 acts antagonistically to N-cadherin, interfering with extracellular N-cadherin adhesion. Mocetinostat, an HDAC inhibitor, inhibits the EMT activator ZEB1, suppressing drug resistance in lung and pancreatic cancer [286].

The reversal of EMT by the induction of MET is the subject of research into counteracting the metastatic potential and stemness associated with EMT induction. Clinical studies have demonstrated the anticancer potential of retinoic acid. The compound has shown efficacy in reversing EMT in BC and paclitaxel-resistant colon cancer cells, reducing tumor cell motility both in vitro and in vivo [292,293]. Aside from retinoic acid, drugs related to epigenetics have surfaced as potential inducers of the MET program in preclinical models [286,294]. Additionally, compounds like metformin and curcumin have shown potential in inhibiting EMT and reversing drug resistance [6,295]. However, it is important to consider the potential adverse effects of these methods. EMT and EMT-TFs play critical roles in normal physiological processes such as wound healing and stem cell homeostasis, so disrupting these processes may have unintended consequences. Furthermore, treatments that promote MET may paradoxically increase tumor metastasis. A careful assessment of side effects and a deeper understanding of the complex role of EMT in cancer are critical to developing effective treatments [6].

### 7.3. Challenges and Opportunities in Translating EMT [Table 1]

Understanding the role of EMT in different cancer types is crucial for effectively targeting the process. Cells undergoing partial EMT display a mixed epithelial-mesenchymal phenotype, which facilitates cancer invasion, spread, and metastasis [296]. Unravelling the full spectrum of EMT morphology and cell-state transitions may improve our understanding of tumor heterogeneity, growth, invasion, metastasis, and drug resistance [2]. EMT contributes to immunosuppression in the tumor microenvironment by activating EMT-TFs, leading to the accumulation of immunosuppressive cells. Combining immunotherapy targeting immunosuppressive cells with immune checkpoint inhibitors is a promising approach to cancer treatment. For example, since EMT is associated with immune suppression, anti-EMT therapy could be used prior to immunotherapy to sensitise the tumor to the treatment. Further research into the role of EMT in tumor development is essential to develop improved treatments that target the EMT process and improve therapy outcomes [2,296].

**Table 1:** Challenges in EMT for cancer therapy.

Challenges	
Complexity of EMT	EMT involves intricate molecular mechanisms and signaling pathways, making it difficult to pinpoint specific targets for therapeutic intervention [6]
Biomarker identification	A challenge in identifying universal biomarkers is the heterogeneity of EMT across cancer types and within individual tumours [6].
Therapeutic resistance	EMT resistance spans chemotherapy, radiotherapy, and more, impeding treatment efficacy at various levels. Common mechanisms of drug resistance include enhanced drug efflux and evasion of apoptosis or necrosis. For instance, SLUG and SNAIL can thwart treatment-triggered apoptosis by disrupting p53 function or suppressing the tumor-suppressor PTEN [2].
Limited clinical validation	Numerous EMT-targeted therapies showing promise in preclinical studies await thorough clinical validation [8].
Adverse effects of anti-EMT treatment	Anti-EMT treatment may have the opposite effect than originally expected. Because EMT plays very important physiological roles, targeting EMT not only affects tumour-cell subpopulations but can also have a negative impact on normal cells [20].

## 8. Future Directions and Challenges

### 8.1. Identification of Novel Regulators and Therapeutic Targets for EMT Inhibition

EMT-induced phenotypic changes enable cancer cells to acquire stem-like properties, evade immune surveillance, and resist cytotoxic therapies, ultimately leading to treatment failure and disease relapse. Thus, identifying novel regulators of EMT is crucial for the further development of therapies. One of the possible regulators is long pentraxin-3 (PTX3), mentioned back in 2013. The overexpression of PTX3 causes melanoma cells to return to their epithelial form, which would suggest that low levels of PTX3 are an unfavorable prognostic factor in melanoma [297]. In contrast, a recent study reported that PTX3 is highly expressed in gliomas compared to normal brain tissue. PTX3 promotes the transcription and secretion of periostin (POSTN), which triggers activation of the MAP-ERK, which promotes EMT-like processes in glioma cells. Thus, the pathway blockage of PTX3-POSTN-MAPK may be beneficial in patients with gliomas [298].

Another study shows that targeting three genes, HGF, PTX3, and S100P, or their associated pathways, could be a promising strategy for inhibiting EMT and combating lung adenocarcinoma (LUAD). In patients with LUAD, there was a significant downregulation of PTX3 and HGF genes, while S100P expression was notably upregulated. HGF and PTX3 can induce the transition of epithelial cells [299].

In patients with pancreatic ductal adenocarcinoma (PAAD), it has been noted that the triggering of the EMT process can be caused by the Circular RNA particle named circ_0092314. The suggested model shows that circ_0092314 can act as sponges for miR-671, thereby increasing S100P expression, which induces the EMT process. Molecules such as circ_0092314, miR-671, and S100P are other potential therapeutic targets in PAAD patients [300].

The research paper focused on the effects of diallyl trisulfide (DATS) on hypoxia-induced gene and protein expressions and showed that targeting HIF-1α genes in BC can inhibit EMT and metastasis, especially under hypoxic conditions. HIF-1α expression is linked to BC metastasis, and inhibiting HIF-1α activity or expression can potentially hinder EMT progression. DATS effectively inhibits HIF-1α transcriptional activity and consequently inhibits EMT. In addition, inhibiting thioredoxin (Trx-1) expression may suppress HIF-1α activity. Further validation and additional investigation of the proposed mechanisms are required to understand how DATS affects HIF-1α transcription or Trx-1 protein [301].

Another study focused on Trx-1 and glucose-6-phosphate dehydrogenase (D6PD) in colorectal cancer, showing that the downregulation of Trx-1 inhibits EMT induced by glucose deprivation. In addition, concomitant downregulation of D6PD can reverse the migration, invasion, and EMT process induced by glucose deprivation. The combined inhibition of Trx-1, G6PD, and glycolysis induces anti-tumor effects in colorectal cancer cells [302].

### 8.2. Integration of Multi-Omics Data for a Comprehensive Understanding of EMT Dynamics

The term multi-omics conceals a broad approach to understanding cellular processes. It includes areas such as genomics, transcriptomics, proteomics, or metabolomics, allowing for correlation analysis between various molecular levels. This holistic approach enables a profound analysis of the targeted cells and the impact of processes like EMT on investigated cells. It has been revealed that the overexpression of KIF18B, a microtubule motor protein that facilitates chromosomal separation and positioning during the process of cell division, in nasopharyngeal carcinoma may be a predictive biomarker for therapeutic response in chemotherapy and immunotherapy. This revelation was possible by correlation between EMT markers, ITGA6, VIM ZEB1/2, and various types of data, such as transcriptomic, spatial transcriptomic, and genomics [303].

The research paper focused on an EMT-related gene FOXM1, which is a potential predictive cancer biomarker in clear-cell renal carcinoma (ccRCC). It shows, through gene-expression data, that 644 out of 704 EMT-related genes in the early stages of ccRCC gene expression are already altered. Furthermore, the study revealed that compared to the early stages, the gene expression of late-stage cancers was completely reversed. This is a very important aspect, as targeted therapy at a particular date of cancer-stage diagnosis will no longer be effective in cancers of the same type at later stages. It is important to always correlate the data obtained with the clinical data and not to refer to only one assumption at a particular level of disease progression [304].

The study about leucine zipper-like transcriptional regulator 1 (LZTR1) presented that LZTR1 deficiency, both in vitro and in vivo, induces EMT and ECM deposition, which contributes to tumor progression and metastasis. Furthermore, the multi-omics approach proves to us that a multi-level investigation through differential gene-expression analysis (DEA) and proteomic analysis allows us to link more reliably the factor–LZTR1 deficiency, which triggers EMT-related proteins [305].

It is noteworthy that by combining spatio-temporal approaches and multi-omics together, we can further enhance our knowledge of the mutual interweaving between various intracellular and extracellular pathways. Additionally, we should combine other non-omic methods in further investigations by combining multi-omic techniques with other more accessible methods, such as ELISA and qPCR [306].

### 8.3. Development of Innovative Therapeutic Strategies to Target EMT-Associated Processes

Studies highlight that due to the evolving understanding of metastasis of various cancer types, which exhibit a distinct reliance on EMT-TFs and effector requirements to initiate EMT and partial EMT, systemic research is needed across diverse in vivo tumor models, which would allow for the understanding of the whole cascade of processes in tumor cells. Furthermore, this information would help in future experiments to determine whether EMT is required for metastasis to occur and whether, in general, changes in EMT in a particular disease are important for the effective elimination of defective cancer cells. Only then will it be possible to definitively answer the question of whether EMT is crucial for metastasis in different [307].

A recent study draws attention to the analysis of rare samples such as CTCs and extracellular vesicles (EVs), which are potential points of interest as biomarkers in the diagnosis and monitoring of cancer. New technologies like microfluidic devices enhance the possibility of isolation and characterization of these membranous samples [306].

One of the new promising therapies is the usage of selenium in the inhibition of histone demethylase; more specifically, lysine (K)-specific demethylase 6B (JMJD3) in cervical cancer cells. The study showed that the blockage of this molecule leads to reverse EMT, including monitoring markers like CDH2, VIM, ZEB1, and ZEB2. It is worth noting that those cells also undergo the promotion of apoptosis and induction of the G2 or M phase [308].

## 9. Conclusions

EMT presents significant promise in revolutionizing the paradigms of cancer diagnosis and treatment. EMT is characterized by abrupt, system-wide shifts triggered when external conditions exceed specific thresholds. This underscores the necessity of evaluating EMT not just at the molecular level but from a broader, system-oriented perspective, considering the pivotal role of the microenvironment in these transitions. A holistic, context-specific approach is crucial for a comprehensive understanding of EMT as a critical transition.

Insights into EMT mechanisms enable the development of innovative strategies to advance early detection, improve prognostic accuracy, enhance treatment outcomes, and personalize care for cancer patients. Key molecular markers of EMT, such as E-cadherin, N-cadherin, vimentin, fibronectin, SNAIL, SLUG, and TWIST, reflect the transition from an epithelial to a mesenchymal phenotype, influencing cell behavior and metastatic potential. Multi-omics approaches that correlate EMT markers across molecular levels can identify predictive biomarkers like KIF18B and FOXM1. The need for multi-level investigations is further emphasized by the role of LZTR1 deficiency in EMT-related processes, underscoring the importance of a comprehensive understanding of EMT dynamics.

Cells undergoing partial EMT exhibit a mixed phenotype, aiding cancer invasion, spread, and metastasis. Unraveling the full spectrum of EMT transitions can improve our understanding of tumor heterogeneity, growth, invasion, metastasis, and drug resistance. EMT also contributes to immunosuppression in the tumor microenvironment, leading to the accumulation of immunosuppressive cells. Combining immunotherapy targeting these cells with immune checkpoint inhibitors is a promising treatment approach. Anti-EMT therapy could sensitize tumors to immunotherapy, highlighting the importance of further research into EMT’s role in tumor development to develop improved treatments and enhance cancer outcomes.

Systemic research encompassing diverse tumor models is essential to elucidate the role of EMT in metastasis across different cancer types. Rare samples such as CTCs and EVs emerge as potential biomarkers for cancer diagnosis and monitoring. Additionally, the potential therapeutic application of selenium in inhibiting JMJD3 in cervical cancer cells shows promise in reversing EMT and inducing apoptosis. These coherent strategies collectively advance our comprehension and management of EMT-associated processes in cancer.

## Data Availability

No new data were created or analyzed in this study.

## Abbreviations

α-SMA	Smooth muscle actin
ACC	Acetyl-CoA carboxylase
Asn	Asparagine
ASNS	Asparagine synthetase
BC	Breast cancer
BFAL1	Bacteroides fragilis lncRNA1
BRAFi	B-Raf Protein Kinase Inhibitors
CAAs	Cancer-associated adipocytes
CAFs	Cancer-associated fibroblasts
ccRCC	Clear cell renal carcinoma
COLI	Collagen-I
COLIV	Collagen-IV
CSCs	Cancer stem cells
CtBP	C-terminal Binding Protein
D6PD	6-Phosphogluconate Dehydrogenase
DATS	Diallyl trisulfide
DDR	Discoidin domain receptor
DDR2	Discoidin domain receptor 2
dNTPs	Deoxyribonucleoside triphosphates
EGF	Epidermal growth factor
EGFRs	Surface receptors for EGF
EMP	E/M plasticity
EMT	Epithelial-mesenchymal transition
ENAH	Enabled Homolog
ERK1/2	Extracellular signal–regulated kinase1 and 2
ERRα	Estrogen-related receptor α
ESRP	Epithelial splicing regulatory protein
Evs	Extracellular vesicles
EZH2	Enhancer of Zeste Homolog 2
FAK	Focal adhesion kinase
FASN	Fatty acid synthase
FBP1	Fructose-1,6-bisphosphatase 1
FGF	Fibroblast growth factor
FGFR2IIIc	Epithelial-Type IIIb isoform to the mesenchymal-like IIIc isoform
FGFRs	FGF receptors
Gln	Glutamine
GRHL2	Grainyhead-like 2
GLS1	Glutaminase 1
GSH	Glutathione
H3K27ac	Histone H3 acetylated at lysine 27
H3K4Me3	H3 lysine 4 trimethylation
H3K36Me3	H3 lysine 36 trimethylation
HCC	Hepatocellular carcinoma
HDACs	Histone Deacetylases
HIFs	Hypoxia-inducible factors
HGF	Hepatocyte growth factor
HNSCC	Head-and-neck squamous cell carcinoma
HREs	Hypoxia response elements
iCAF	Inflammatory cancer-associated fibroblasts
Ig	Immunoglobulin
IGF-1R	Insulin-like growth factor-1R
IKK	IκB kinase
iNOS	Induced nitric oxide synthase
JMJD3	Lysine (K)-specific demethylase 6B
JNK	Jun N-terminal kinase
lncRNAs	Long non-coding RNAs
LOCKs	Large Organized Chromatin K9-modified Domains
LOX	Lysyl oxidase
LUAD	Lung adenocarcinoma
LZTR1	Leucine zipper-like transcriptional regulator 1
MALAT1	Metastasis-associated lung adenocarcinoma transcript 1
MAPKi	MAP Kinase Inhibitors
Mbd3/NuRD	Methyl CpG-binding domain/3 Mi-2/nucleosome remodeling and deacetylase complex
MCP-1	Monocyte chemoattractant protein-1
MDSCs	Myeloid-derived suppressor cells
MET	Mesenchymal-epithelial transition
myCAFs	Myofibroblastic cancer-associated fibroblasts
NF-κB	Nuclear factor κ-light-chain-enhancer of activated B cells
NICD	NOTCH intracellular domain
OPG	Osteoprotegerin
OS	Overall survival
PAAD	Pancreatic ductal adenocarcinoma
PC	Prostate cancer
PFS	Progression-free survival
PKA	Protein kinase A
POSTN	Periostin
pO2	Oxygen partial pressure
PPP	Pentose phosphate pathway
PRC2	Polycomb Repressive Complex 2
PTX3	Long pentraxin-3
pVHL	Von Hippel-Lindau protein
PyM	Pyrimidine metabolism
ROS	Reactive oxygen species
SCD	Stearoyl-CoA desatuRase
SGOC	Serine/glycine one-carbon
SNHG15	Small nucleolar RNA host gene 15
SPARC	Secreted protein acidic and rich in cysteine
SRSF1	Serine/arginine-rich Splicing Factor 1 protein
SSP	Serine synthesis pathway
SUZ12	Suppressor of Zeste 12
TACE	TNF-α converting enzyme
TAK1	Transforming growth factor-activating kinase 1
TCF2	Transcription factor 2
TGFβ	Transforming growth factor β
TME	Tumor microenvironment
TMEM	Microenvironment metastasis
TNF-α	Tumor necrosis factor α
UPR	Unfolded Protein Response
